# Sequential Inhibition of PARP and BET as a Rational Therapeutic Strategy for Glioblastoma

**DOI:** 10.1002/advs.202307747

**Published:** 2024-06-19

**Authors:** Xin Peng, Xin Huang, Shaolu Zhang, Naixin Zhang, Shengfan Huang, Yingying Wang, Zhenxing Zhong, Shan Zhu, Haiwang Gao, Zixiang Yu, Xiaotong Yan, Zhennan Tao, Yuxiang Dai, Zhe Zhang, Xi Chen, Feng Wang, Francois X. Claret, Moshe Elkabets, Ning Ji, Yuxu Zhong, Dexin Kong

**Affiliations:** ^1^ Tianjin Key Laboratory of Technologies Enabling Development of Clinical Therapeutics and Diagnostics School of Pharmacy Tianjin Medical University Tianjin 300070 China; ^2^ Key Laboratory of Immune Microenvironment and Diseases (Ministry of Education) International Joint Laboratory of Ocular Diseases (Ministry of Education) Tianjin Medical University Tianjin 300070 China; ^3^ Department of Systems Biology the University of Texas MD Anderson Cancer Center Houston TX 77030 USA; ^4^ State Key Laboratory of Toxicology and Medical Countermeasures Beijing Institute of Pharmacology and Toxicology Beijing 100850 China; ^5^ Department of Neurosurgery the Affiliated Drum Tower Hospital School of Medicine Nanjing University Nanjing 210008 China; ^6^ Tianjin Key Laboratory of Ophthalmology and Visual Science Tianjin Eye Institute Tianjin Eye Hospital Tianjin 300020 China; ^7^ State Key Laboratory of Medicinal Chemical Biology Nankai University Tianjin 300071 China; ^8^ Department of Genetics School of Basic Medical Sciences Tianjin Medical University Tianjin 300070 China; ^9^ The Shraga Segal Department of Microbiology Immunology and Genetics Faculty of Health Sciences Ben‐Gurion University of the Negev Beer‐Sheva 84105 Israel; ^10^ National Clinical Research Center for Cancer Tianjin's Clinical Research Center for Cancer Key Laboratory of Cancer Prevention and Therapy Tianjin Medical University Cancer Institute and Hospital Tianjin 300060 China; ^11^ Department of Pharmacy Tianjin Medical University General Hospital Tianjin 300052 China

**Keywords:** BET, cell cycle, DNA damage, glioblastoma, PARP

## Abstract

PARP inhibitors (PARPi) hold substantial promise in treating glioblastoma (GBM). However, the adverse effects have restricted their broad application. Through unbiased transcriptomic and proteomic sequencing, it is discovered that the BET inhibitor (BETi) Birabresib profoundly alters the processes of DNA replication and cell cycle progression in GBM cells, beyond the previously reported impact of BET inhibition on homologous recombination repair. Through in vitro experiments using established GBM cell lines and patient‐derived primary GBM cells, as well as in vivo orthotopic transplantation tumor experiments in zebrafish and nude mice, it is demonstrated that the concurrent administration of PARPi and BETi can synergistically inhibit GBM. Intriguingly, it is observed that DNA damage lingers after discontinuation of PARPi monotherapy, implying that sequential administration of PARPi followed by BETi can maintain antitumor efficacy while reducing toxicity. In GBM cells with elevated baseline replication stress, the sequential regimen exhibits comparable efficacy to concurrent treatment, protecting normal glial cells with lower baseline replication stress from DNA toxicity and subsequent death. This study provides compelling preclinical evidence supporting the development of innovative drug administration strategies focusing on PARPi for GBM therapy.

## Graphical abstract

Glioblastoma, a challenging cancer type, faces limitations with current therapies. PARP inhibitors show therapeutic promise but continuous administration presents adverse effects. This study, driven by transcriptomic and proteomic analysis, unveils the synergistic potential of sequentially combining Rucaparib with Birabresib in enhancing glioblastoma treatment based on the precise manipulation of replication stress. These findings suggest a promising direction for glioblastoma treatment strategies.

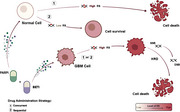

## 1 Introduction

Glioblastoma (GBM) is one of the most aggressive and lethal forms of brain cancer, characterized by rapid growth and resistance to treatment.^[^ ^1^ ^]^ It predominantly affects the brain's supportive tissue, with a complex and heterogeneous tumor environment.^[^ ^2^ ^]^ Despite advancements in neuro‐oncology, the prognosis for GBM remains poor, with a median survival of ≈15 months postdiagnosis.^[^ ^3^ ^]^ The intricate nature of GBM, coupled with its robust defensive mechanisms, poses significant challenges in developing effective therapeutic strategies.^[^ ^4^ ^]^ Consequently, there is an urgent need for innovative approaches and comprehensive studies to understand the molecular and cellular dynamics of GBM, and to identify novel targets and optimize therapeutic interventions for improving patient outcomes.^[^ ^5^ ^]^ In this context, exploring the synergistic effects of various drug combinations and elucidating the underlying mechanisms hold promise in advancing GBM treatment paradigms.^[^ ^6^ ^]^

Poly (ADP‐ribose) polymerase (PARP) plays a crucial role in maintaining genomic stability. The primary functions of PARP encompass repairing single‐strand breaks (SSB), stabilizing replication forks, and addressing replication stress.^[^ ^7^ ^]^ PARP1 facilitates SSB repair by undergoing auto PARylation, which allows it to dissociate from DNA and recruit other repair proteins to the site of damage.^[^ ^8^ ^]^ PARP inhibitors (PARPi) prevent auto PARylation and trap PARP on DNA, which obstructs the progression of replication forks and can escalate to the formation of double‐strand breaks (DSB).^[^ ^9^ ^]^ To counteract DSB and maintain genomic integrity, various mechanisms have evolved to repair DSB, with homologous recombination (HR) being the sole high‐fidelity DSB repair process.^[^ ^10^ ^]^ The ability of PARPi to induce DSB can be lethal for HR‐deficient cells, but not for HR‐proficient ones, representing synthetic lethality.^[^ ^11^ ^]^ The expanding application of PARPi is evident, with more than 20 clinical trials using PARPi for GBM, highlighting their promising role in enhancing GBM treatment (clinicaltrials.gov, accessed May 2024).

Bromodomain and Extra‐Terminal domain (BET) proteins, including BRD2, BRD3, BRD4, and BRDT, are vital regulators of gene expression. They recognize acetylated lysine residues on histone tails and influence chromatin structure and function.^[^ ^12^ ^]^ These proteins are integral to the transcriptional machinery, impacting various cellular processes such as cell cycle progression, apoptosis, and inflammation.^[^ ^13^ ^]^ The aberrant expression or dysfunction of BET proteins, particularly BRD4, is significantly implicated in GBM, emphasizing their role in disease progression.^[^ ^14^ ^]^ Previous reports have demonstrated that BRD4 can regulate the expression of crucial genes involved in HR repair, leading to HR deficiency (HRD) and inducing synthetic lethality with PARPi.^[^ ^15^ ^]^ This discovery marked the first linkage between BET proteins and PARPi, and subsequent research has primarily centered on the mechanisms by which BET inhibitors (BETi) modulate HR to sensitize cells to PARPi.^[^ ^16^ ^]^

Clinical studies have already incorporated BETi into the treatment of GBM.^[^ ^17^ ^]^ In this study, we selected the BET inhibitor (BETi) Birabresib, which is under clinical trials for GBM (clinicaltrials.gov, accessed May 2024). Birabresib is a potent BET bromodomain inhibitor with EC50 ranging from 10 to 19 nm for BRD2, BRD3, and BRD4. It has shown strong in vitro and in vivo antitumor activity across various xenograft models.^[^ ^18^ ^]^ For the first time, we conducted a comprehensive analysis of its impact on gene and protein expression patterns in GBM cells through unbiased transcriptomic and proteomic sequencing. We found that BET inhibition not only significantly impacts HR repair as previously reported,^[^ ^15^, ^16^ ^]^ but profoundly alters the processes of DNA replication and cell cycle progression in GBM cells. These observations strongly suggest a potential deeper level of synergistic interaction with PARPi, revealing a broader and greater depth of effects than previously understood. Inspired by these findings, we aim to explore the combined therapeutic strategy of Birabresib enhancing the cytotoxic effect of PARPi on GBM and to unveil the underlying mechanisms.

## 2 Result

### 2.1 RNA‐seq and Quantitative Proteomics Reveal Birabresib's Impact on GBM Cell Gene and Protein Expression Profiles

**Figure 1:**
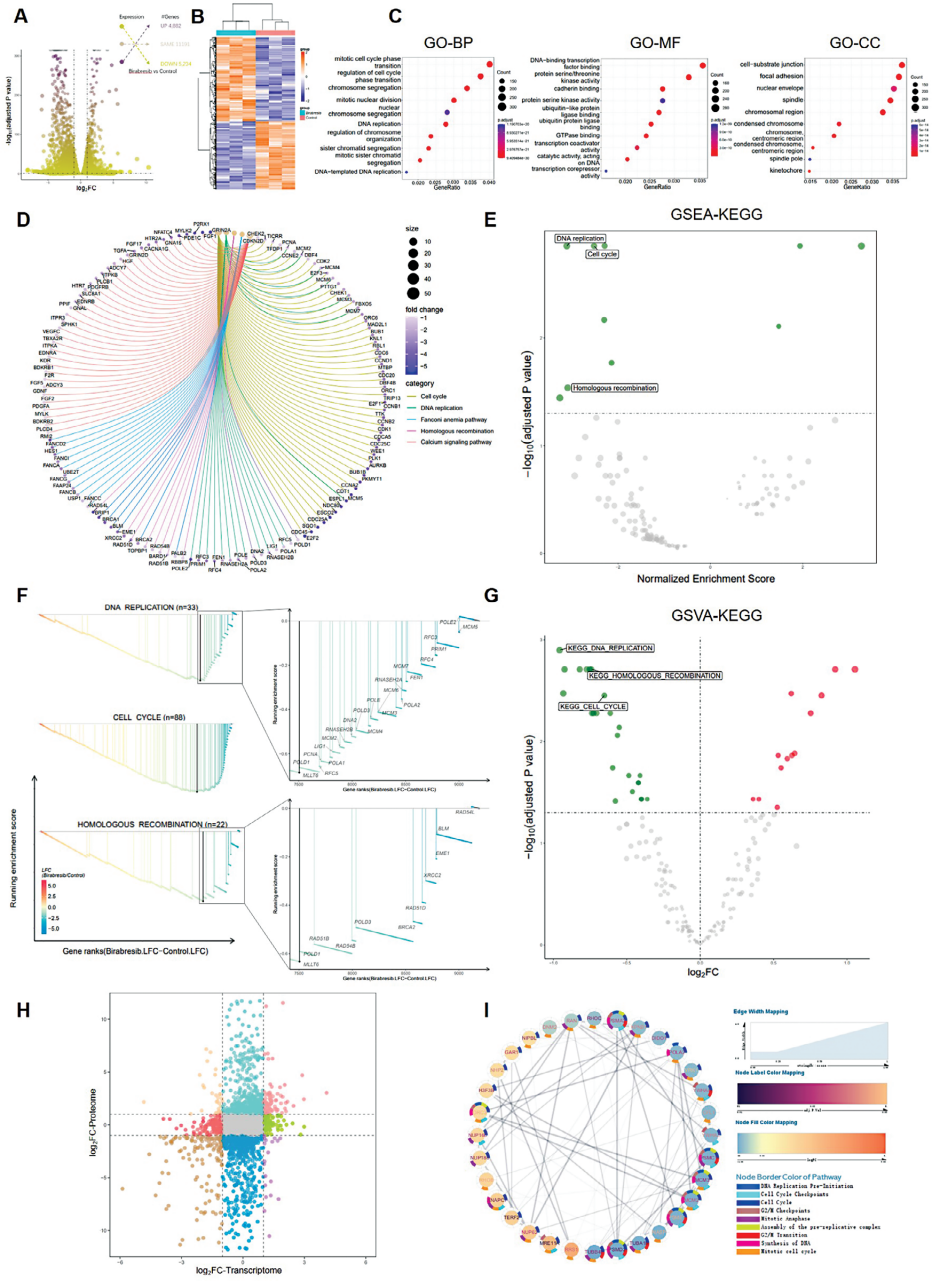
Birabresib Treatment Alters Gene and Protein Expression. U87 cells were treated with Birabresib (0.5 µm) or DMSO (Control) for 24 h and subjected to RNA‐Seq and quantitative proteomics sequencing. A) Volcano plot displaying global gene expression with log‐fold changes in gene expression and adjusted *p*‐values. The horizontal dashed line indicates an adjusted *p*‐value of 0.05. B) Heatmap showing differentially expressed genes, with FPKM values converted to Z‐scores and subjected to clustering analysis. C) Bubble chart illustrating the enrichment of differentially expressed genes mapped to GO (Gene Ontology) pathways, including GO‐BP (Biological Process), GO‐BP (Biological Process), and GO‐CC (Cellular Component). Adjusted *p*‐values are represented by color variations, and the size of the bubbles corresponds to the number of genes in each term. D) Circular gene‐concept network diagram presenting enriched KEGG pathways and gene networks. The size of the bubbles at each pathway corresponds to the number of associated genes, with different pathways represented by different colors. Log‐fold changes in gene expression are depicted by color variations. E) Scatter plot showing gene set enrichment analysis (GSEA) of differentially expressed genes mapped to KEGG pathways, including normalized enrichment scores and adjusted *p*‐values. The horizontal dashed line indicates an adjusted *p*‐value of 0.05. F) Bar chart displaying selected pathways from the KEGG gene set enrichment analysis, including enrichment scores and gene rankings. Log‐fold changes in gene expression are represented by color variations, with top‐ranked genes magnified on the right. G) Volcano plot illustrating gene set variation analysis of genes mapped to KEGG pathways, including log‐fold changes in enrichment scores and adjusted *p*‐values. The horizontal dashed line indicates an adjusted *p*‐value of 0.05. H) Nine‐quadrant diagram comparing the log‐fold change values of transcriptome and proteome. Color‐coded genes with the adjusted *p*‐value <0.05 (*n* = 3 per group for proteomic profiling; *n* = 3 for RNA‐seq). I) Protein‐protein interaction network diagram of nine Reactome pathways related to DNA replication and cell cycle. Each protein's pathway affiliation is reflected in the color of the node border, with each color corresponding to a specific pathway. Log‐fold changes in protein expression are represented by color variations in a clockwise decreasing manner. Adjusted P‐values for protein expression are depicted by label color variations. Composite scores for protein interactions are represented by the thickness and transparency of the edges.

To thoroughly investigate the biological impact of BETi Birabresib on GBM cells, we conducted RNA‐Seq and quantitative proteomics sequencing using U87 cells treated with Birabresib. First, at the transcriptome level, we identified 4882 upregulated genes and 5234 downregulated genes (**Figure** **1**A,B). Gene Ontology (GO) enrichment analysis revealed that genes related to “mitotic cell cycle phase transition,” “DNA replication,” etc., ranked in the top ten positions, with their molecular functions and cellular components closely related to “DNA‐binding transcription factor binding” and “chromosomal region” respectively (*p* < 10^−8^, Figure 1C). Similar results were obtained in other databases such as the Kyoto Encyclopedia of Genes and Genomes (KEGG) and Hallmark (Figure S1A,B, Supporting Information). Enriched KEGG pathways were displayed using a Circular gene‐concept network (Figure 1D). Gene Set Enrichment Analysis (GSEA) yielded very consistent results (Figure 1E,F; Figure S1C, Supporting Information), with “DNA replication” and “cell cycle” emerging as the top two processes that were significantly inhibited by Birabresib, followed by “homologous recombination repair” (NES were −3.11, −2.52, −2.29 respectively; *p* values were 0.00005, 0.00005, 0.0019 respectively). Gene Set Variation Analysis (GSVA) also resulted in the same findings (Figure 1G; Figure S1D, Supporting Information), and a heatmap displayed the gene expression changes in related pathways (Figure S1E, Supporting Information). To verify the accuracy of the sequencing results, we analyzed the expression of several key genes using qRT‐PCR and found a dose‐dependent decrease with Birabresib treatment, consistent with the sequencing results (MCM3, CHEK1, CHEK2, WEE1, TOPBP1, BRCA1, BRCA2, RAD51, and RAD54B) (Figure S1F, Supporting Information).

At the proteome level, we discerned 473 upregulated proteins and 426 downregulated proteins (Figure S2A,B, Supporting Information). Protein–protein interaction (PPI) analysis displayed significantly altered proteins (Figure S2C, Supporting Information). A correlation analysis revealed substantial concordance between the proteomic and transcriptomic data (Figure 1H and Figure S2D,E, Supporting Information). The pathways enriched in the transcriptome were similarly predominant at the proteomic level (Figure 1I). The unbiased sequencing outcomes depicted the pronounced phenotypes associated with impaired DNA replication and repair induced by Birabresib, leading us to hypothesize that Birabresib harbors the potential to augment the efficacy of PARPi against GBM.

### 2.2 Birabresib Synergizes with PARP Inhibitors to Suppress GBM

**Figure 2:**
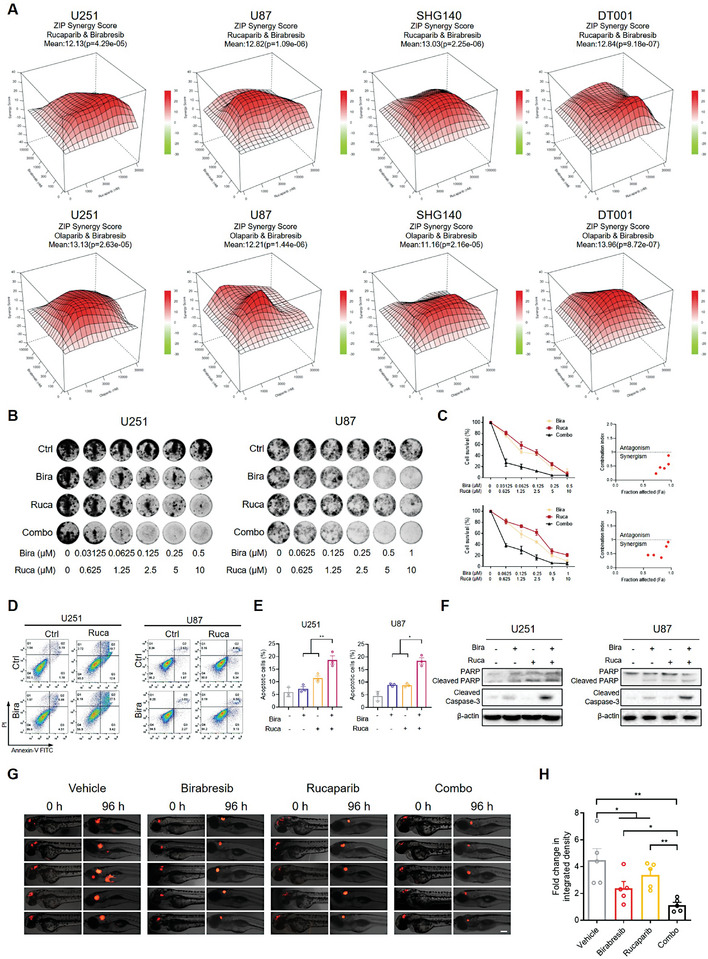
Synergistic Suppression of GBM by Birabresib and PARP Inhibitors. A) The ZIP model synergy analysis was performed in established cell lines of GBM (U251 and U87), and patient‐derived primary GBM cell lines (SHG140 and DT001). GBM cells were treated with increasing concentrations of Olaparib/Rucaparib and Birabresib for 96 h, then the inhibition rate of tumor cell growth was calculated. The results are presented as a ZIP synergy score with the dose‐response matrix. B) Clonogenic assays for GBM cells treated with Birabresib and/or Rucaparib for 72 h, which were allowed to recover for 10–15 days and then subjected to crystal violet staining. C) Quantification of B), the light absorbance at 570 nm was measured after incubation with 1% SDS for 3 h. Cell survival (%) is expressed as a percentage of the control; CIs were calculated with CalcuSyn. CI ≤ 0.9 represents synergism, 0.9 < CI ≤ 1.1 represents additivity, and CI > 1.1 represents antagonism. D,E) Apoptosis assays for GBM cells treated with 0.5 µm Birabresib and/or 20 µm Rucaparib for 48 h. Apoptosis was detected by Annexin V/PI staining. FACS quantification of the total apoptotic cell population, including Annexin V+/PI− early apoptotic cells and Annexin V+/ PI+ late apoptotic cells. F) Western blot analysis of Cleaved Caspase‐3 and PARP in U251 and U87 cells treated with Birabresib and/or Rucaparib for 48 h. G) Fluorescence image of embryos in the orthotopic zebrafish model. After transplantation of 50–100 U87‐RFP cells, the injected embryos were transferred to a 96‐well plate containing the drugs and incubated for 96 h. The embryos were imaged under a fluorescence microscope to evaluate tumor growth. Scale bar, 100 µm. H) The fluorescence intensity of different groups, as determined at the experimental endpoint (96 h) and expressed as fold change in integrated density. Graphs are presented as the mean ± SEM from three independent experiments; *p*‐values were determined using a two‐tailed unpaired Student's *t*‐test; ^*^ *p* < 0.05; ^**^ *p* < 0.01.

Driven by the omics data, we initially investigated the effects of the combined administration of Birabresib and PARPi in established GBM cell lines and patient‐derived primary GBM cells. The ZIP synergy model indicated a strong synergistic interaction between Birabresib and PARPi, with average scores in all four cell lines exceeding 10. The 3D surface plots revealed regions of synergy with average ZIP synergy scores, accompanied by a dose‐response matrix (**Figure** **2**A; Figure S3A–D, Supporting Information). To further substantiate these findings, we employed clonogenic assays, which confirmed the synergistic interaction between Birabresib and PARPi, evidenced by CI values less than 1 (Figure 2B,C; Figure S4A,B, Supporting Information). Consistently, apoptosis assays demonstrated that the combination of Birabresib and PARPi induced significantly higher levels of apoptosis compared to either agent alone (Figure 2D,E), associated with increased cleavage of Caspase‐3 and PARP (Figure 2F). To rule out potential off‐target effects of Birabresib, we employed siRNA to knock down BRD4 (Figure S4C,D, Supporting Information) and another BETi, JQ1 (Figure S4E, Supporting Information), both of which could mimic the effects of Birabresib. Given the significant in vitro results, we further transplanted fluorescently labeled U87 cells into the zebrafish cranium to allow characterization of the tumor growth state.^[^ ^19^ ^]^ We observed that both Birabresib and PARPi, when administered a single agent, inhibited tumor growth in the zebrafish cranium to a certain extent, but their combination manifested a potent inhibitory effect significantly greater than single‐drug treatment (Figure 2G,H). These results convincingly demonstrate the synergistic anti‐GBM effects of Birabresib and PARPi.

### 2.3 Birabresib Promotes Cell Entry into Mitosis and Inhibits the DNA Damage Repair Response in PARPi‐Treated GBM Cells

**Figure 3:**
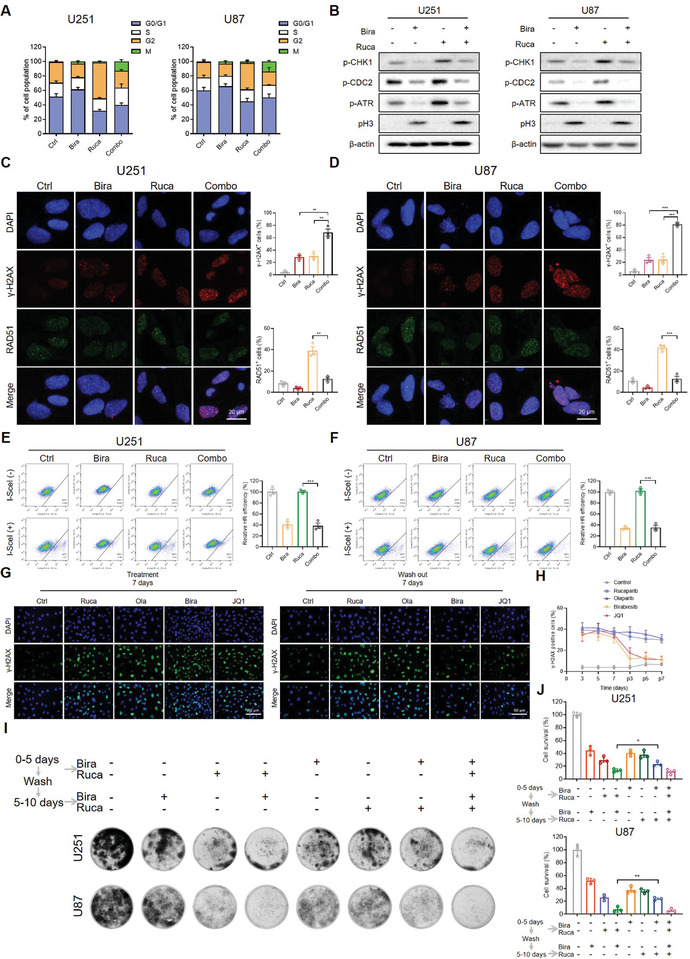
Birabresib and PARPi Alter Cell Cycle Progression and DNA Damage Repair. A) GBM cells were treated with DMSO, 0.5 µm Birabresib, 20 µm Rucaparib, or a combination of both for 24 h, followed by pH3 and PI flow cytometry analysis. B) Expression of the indicated cell cycle checkpoint proteins was examined by Western blot. C,D) GBM cells were subjected to γ‐H2AX and RAD51 foci assays after treatment with Rucaparib, Birabresib, or a combination of both. Representative images and quantification of U251 and U87 cells with positive γ‐H2AX/RAD51 signals (more than 5 foci per cell). Scale bar, 20 µm. E,F) DR‐GFP reporter assays evaluated the effect of Birabresib and Rucaparib on HR repair efficiency in U251 and U87 cells. Relative HR efficiency is expressed as % of the control. G) γ‐H2AX foci assays of U251 cells treated with BETi (0.02 µm Birabresib or 0.01 µm JQ1) and PARPi (1 µm Rucaparib or 1 µm Olaparib). The formation and resolution of γ‐H2AX foci were assessed using immunofluorescence. Scale bar, 50 µm. H) The percentage of γ‐H2AX positive cells (with > 5 foci per cell) is shown at each time point. Representative images are displayed. I) Cells were treated with DMSO, Birabresib, and Rucaparib, a sequential combination, or a concurrent combination, followed by Clonogenic Assays. J) Quantification of I), the light absorbance at 570 nm was measured after incubation with 1% SDS for 3 h. Cell survival (%) is expressed as % of the control. Graphs are presented as the mean ± SEM from three independent experiments; *p*‐values were determined using a two‐tailed unpaired Student's *t*‐test; ^*^ *p* < 0.05; ^**^ *p* < 0.01; ^***^ *p* < 0.001.

Subsequently, we explored the underlying mechanisms by which the combination of Birabresib and PARPi synergistically triggers apoptosis in GBM cells. Cell cycle analysis manifested that the inhibition of PARP led to a G2 phase arrest, while Birabresib facilitated the transition of cells into the M phase (**Figure** **3**A; Figure S5A, Supporting Information). This transition was further validated by the presence of the mitotic marker pH3, as evidenced by Western blot analysis (Figure 3B; Figure S5B, Supporting Information). Simultaneously, the coadministration of Birabresib diminished the Rucaparib‐induced phosphorylation of CHK1 and ATR, to a degree similar to that observed with Birabresib alone, indicating a compromise in the integrity of DNA damage and cell cycle checkpoints. Birabresib's mechanism of inhibiting cell cycle checkpoints plays a pivotal role in this context, and this inhibition is sustained even when combined with PARPi, contributing to their synergistic effect. Correspondingly, the phosphorylated CDC2, indicative of the G2/M checkpoint, was also attenuated with the singular or combined application of Birabresib, illustrating that Birabresib impairs the G2/M checkpoint (Figure 3B; Figure S5B, Supporting Information). Furthermore, the application of Birabresib and Rucaparib markedly amplified the expression and the foci formation of the DNA damage marker γ‐H2AX. The assembly of RAD51 foci, indicative of DNA double‐strand break repair, was notably compromised when Rucaparib was coadministered with Birabresib (Figure 3C,D; Figure S5C, Supporting Information). The expression levels of BRCA1/2 and RAD51 were also diminished, consistent with recent publications (Figure S5E, Supporting Information).^[^ ^15^ ^]^ Moreover, we determined that Birabresib significantly impairs the efficiency of HR repair. Using a DR‐GFP reporter assay, we found that Birabresib reduced HR repair efficiency to ≈30% of normal levels, regardless of whether it was used alone or in combination with Rucaparib (Figure 3E,F; Figure S5E, Supporting Information).

### 2.4 DNA Damage Induced by PARPi Has a Residual Effect

The process of HR repair for DNA damage exhibits inherent latency,^[^ ^20^ ^]^ implying that the inflicted DNA damage in GBM cells by the medication might persist even after the cessation of drug administration. We hypothesized that sequential administration could sustain the therapeutic impact on the tumor while mitigating adverse effects. To explore the enduring nature of DNA damage induced by both drugs, we assessed γ‐H2AX foci on the day 7 postadministration of BETis and PARPis and on the day 7 following drug withdrawal. Our findings revealed that the levels of γ‐H2AX foci remained elevated post‐withdrawal of both PARPis, Olaparib, and Rucaparib, whereas the DNA damage induced by BETi Birabresib and JQ1 showed signs of recovery postwithdrawal (Figure 3G,H). Additionally, we observed that the elevation in Cyclin B1 induced by PARPis persisted for at least the 7 days post‐treatment (Figure S5F, Supporting Information). These observations underscore the more substantial residual effects of PARPi. Following this, we conducted a comparison between two strategies of sequential administration and concurrent administration of Rucaparib and Birabresib. The results indicated that administering Rucaparib prior to Birabresib is more efficacious than the inverse sequence, and its efficacy was comparable to that of concurrent administration (Figure 3I,J). The sequence and timing in sequential administration often significantly influence antitumor effects of the drugs.^[^ ^21^ ^]^ Our results suggested that initiating treatment with PARPi followed by BETi is a potentially more effective strategy.

### 2.5 Sequential Treatment with Rucaparib and Birabresib is Effective In Vitro

**Figure 4:**
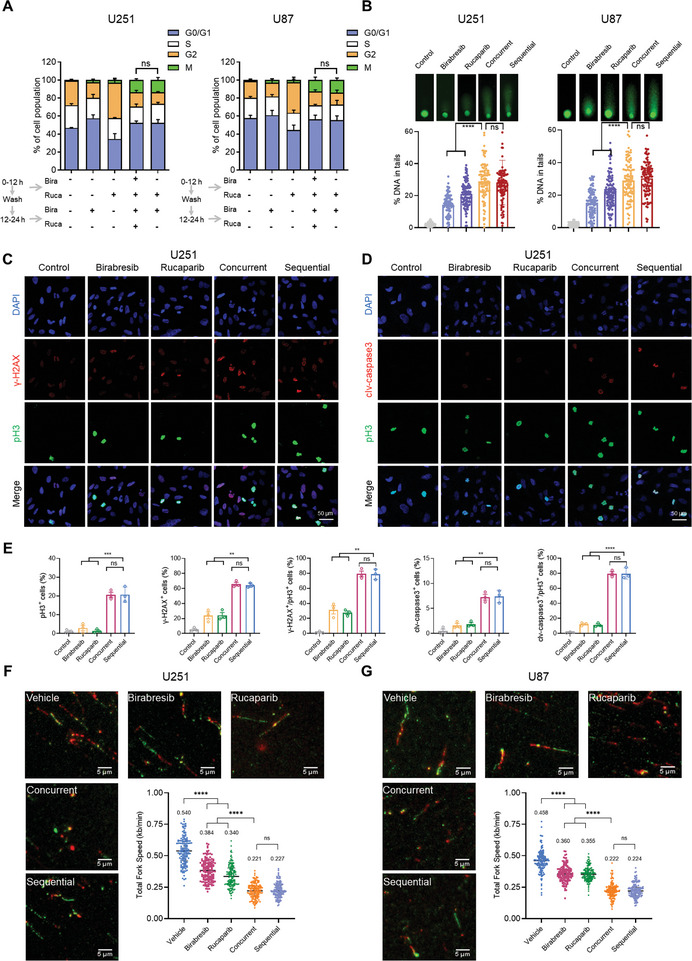
In Vitro Efficacy of Sequential Treatment with Rucaparib and Birabresib. A) GBM cells were treated with DMSO, 0.5 µm Birabresib, 20 µm Rucaparib, sequential, and concurrent treatment, followed by pH3 and PI flow cytometry analysis. B) GBM cells were treated as in (A) and subjected to Comet assay. DNA damage is quantified as a percentage of DNA in the tail. C) Representative images of U251 cells treated as in (A) stained for γ‐H2AX, pH3, and DAPI. Scale bar, 50 µm. D) Representative images of U251 cells treated as in (A) stained for cleaved caspase‐3, pH3, and DAPI. Scale bar, 50 µm. E) Quantitative analysis of (C) and (D) was performed on five photographs from each of the three independent experiments. F,G) Representative images of GBM cells treated as in (A) and subjected to DNA fiber analysis. Scale bar, 5 µm. Mean fork speed (kb min^−1^) is indicated. Graphs are presented as the mean ± SEM from three independent experiments; *p*‐values were determined using a two‐tailed unpaired Student's *t*‐test; ^**^ *p* < 0.01; ^***^ *p* < 0.001; ^****^ *p* < 0.0001.

To gain a more profound understanding of the mechanisms underlying sequential treatment, we investigated its influence on the cell cycle distribution of various GBM cells. In alignment with concurrent treatment, sequential therapy notably mitigated the G2 arrest triggered by Rucaparib and prominently elevated the rate of mitosis (**Figure** **4**A; Figure S6A, Supporting Information). Both sequential and concurrent strategies induced DNA damage to a similar extent (Figure 4B). Foci assays revealed that DSBs (pH3 positive + γ‐H2AX positive) and apoptosis (pH3 positive + Caspase‐3 positive) were present in over 70% of mitotic GBM cells (Figure 4C–E; Figure S6C–F, Supporting Information). The accumulated DNA damage and induced apoptosis can be attributed to the amplified replication stress and the impaired G2 checkpoint resulting from the combination therapy, consequently curtailing the duration available for DNA repair.^[^ ^22^ ^]^ In both sequential and concurrent administration groups, we observed a reduction in replication fork speed compared to single‐agent administration, suggesting an enhancement in replication stress in GBM cells due to the synergistic effect of the drugs, with the intensity being equivalent (Figure 4F,G; Figure S6G, Supporting Information). These findings imply that sequential treatment with PARPi Rucaparib and BETi Birabresib in GBM could potentially achieve therapeutic outcomes comparable to concurrent treatment in terms of efficacy.

### 2.6 Sequential Treatment with Rucaparib and Birabresib Mitigates Toxicity to Normal Glial Cells

**Figure 5:**
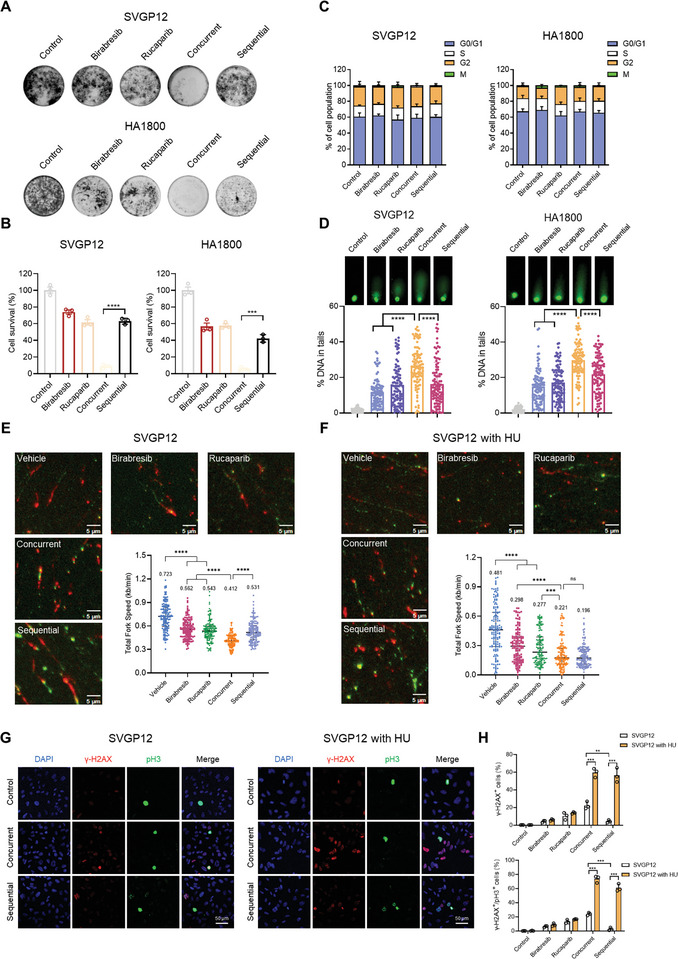
Reduced Cellular Toxicity Observed with Sequential Treatment of Rucaparib and Birabresib. A) Normal glial cells were treated with DMSO, Birabresib, Rucaparib, sequential, and concurrent treatment (as in Figure 3I), followed by Clonogenic Assays. B) Quantification of (A), the light absorbance at 570 nm was measured after incubation with 1% SDS for 3 h. Cell survival (%) is expressed as % of the control. C) Normal glial cells were treated with DMSO, Birabresib, Rucaparib, sequential, and concurrent treatment (as in Figure 4A) and subjected to pH3 and PI flow cytometry analysis. D) Normal glial cells were treated as in Figure 4A and subjected to Comet assay. DNA damage is quantified as a percentage of DNA in the tail. E,F) Normal glial cells were treated as in Figure 4A with or without 50 µm HU for 24 h and subjected to DNA fiber analysis. Scale bar, 5 µm. Mean fork speed (kb min^−1^) is indicated. G,H) Normal glial cells were treated as in Figure 4A with or without 50 µm HU for 24 h and subjected to γ‐H2AX foci analysis. Scale bar, 5 µm. Graphs are presented as the mean ± SEM from three independent experiments; *p*‐values were determined using a two‐tailed unpaired Student's *t*‐test; ^**^ *p* < 0.01; ^***^ *p* < 0.001; ^****^ *p* < 0.0001.

Having demonstrated the considerable efficacy of sequential treatment, we sought to further explore to what extent this approach could alleviate toxicity. We employed clonogenic assays to examine the effects of various treatments on normal glial cell survival. Remarkably, sequential treatment significantly diminished toxicity compared to concurrent treatment, achieving a level of toxicity comparable to that observed with the administration of a single agent (**Figure** **5**A,B).

Next, we investigated the disparate responses of normal glial cells to concurrent and sequential treatments. Intriguingly, neither drug, whether administered alone or in combination, significantly altered the cell cycle of normal glial cells (Figure 5C). In terms of DNA damage, comet assays indicated that the level of DNA damage in the concurrent treatment group was markedly higher than in the sequential treatment group (Figure 5D), implying that the decreased toxicity of sequential treatment may be attributed to its less induction of replication stress compared to concurrent treatment. Subsequently, utilizing the DNA fiber assay, we discerned that normal glial cells exhibited lower inherent levels of replication stress, evidenced by higher replication fork speeds, compared to GBM cells (Figures 4F,G and 5E; Figure S5G, Supporting Information). While concurrent treatment uniformly reduced replication fork speed in both cell types, sequential treatment resulted in a more moderate reduction in normal glial cells (Figure 5E), potentially offering them greater tolerance to the treatment. Further, by artificially inducing replication stress in normal glial cells with hydroxyurea (HU) through nucleotide depletion to mimic the stress levels in GBM cells, we observed that both concurrent and sequential treatments could cause a comparable degree of reduction in replication fork speed (Figure 5F). HU treatment also exacerbated DNA damage caused by sequential treatment (Figure 5G,H). Therefore, the artificially induced higher level of replication stress renders normal glial cells more susceptible to sequential treatment, providing the theoretical basis for employing sequential treatment to maintain efficacy while minimizing toxicity.

### 2.7 Sequential Administration of Rucaparib and Birabresib Maintains Efficacy while with Low Toxicity In Vivo

**Figure 6:**
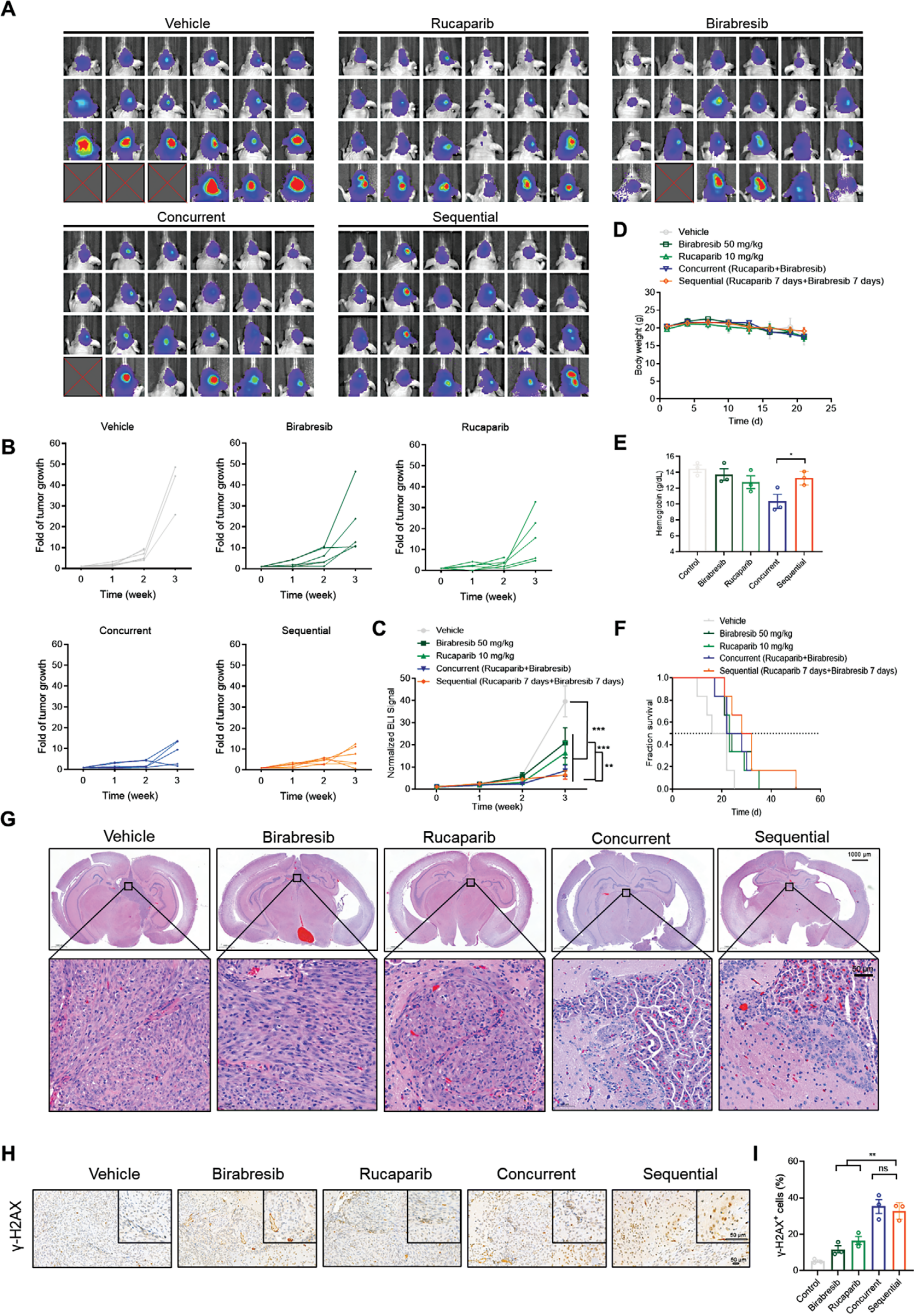
Sequential Administration of Rucaparib and Birabresib Achieves Superior Therapeutic Effects. A) Bioluminescence images of the nude mouse xenograft model mice. Mice were intracranially inoculated with U87‐Luc cells. The localization and intensity of luciferase expression were monitored by in vivo bioluminescence imaging. Following treatment with vehicle, Rucaparib (10 mg kg^−1^), Birabresib (50 mg kg^−1^), concurrent (Rucaparib + Birabresib), or sequential (Rucaparib 7 days + Birabresib 7 days) for 21 days (*n* = 6), animals were assessed using a bioluminescence imaging system. Representative bioluminescent images acquired at the indicated time points are shown. B) Data are shown as a growth curve for each individual tumor. C) The relative radiance on days 0, 7, 14, and 21, which represent tumor size, was determined using the imaging system. Values on day 21 were used for statistical analysis of the difference between any two groups. D) Bodyweight curves during the 21 days of treatment. E) On day 21, peripheral blood was collected from mice in each group to measure hemoglobin levels. F) Kaplan–Meier survival curves of vehicle, vehicle, Rucaparib (10 mg kg^−1^, i.p.), Birabresib (50 mg kg^−1^, i.p.), concurrent (Rucaparib + Birabresib), and sequential (Rucaparib 7 days + Birabresib 7 days) groups. G) Hematoxylin and eosin‐stained section (original magnification 20×) of intracranial U87 mouse xenografts following treatments. H) Representative immunohistochemistry staining for γ‐H2AX in tumor tissues of U87 xenografts. Scale bar, 50 µm. I) Statistical quantification of (H), each point represents the mean values of five images. *p*‐values, two‐tailed unpaired Student's *t*‐test; ^*^ *p* < 0.05; ^**^ *p* < 0.01; ^***^ *p* < 0.001.

Then, we assessed the anti‐GBM efficacy of different administration strategies using the U87 orthotopic mouse tumor model.^[^ ^19^ ^]^ Consistent with findings from the zebrafish model, the combined application of Rucaparib and Birabresib demonstrated superior antitumor activity compared to the individual agents. Crucially, the therapeutic outcomes of sequential administration paralleled those of concurrent administration (**Figure** **6**A–C). Neither administration strategy significantly impacted the body weight of the mice (Figure 6D) or exhibited discernible effects on major organs (Figure S7, Supporting Information). However, the survival period for sequential administration was slightly higher compared to concurrent administration (Figure 6E). Furthermore, concurrent administration of PARPi is often associated with hematological toxicity; notably, the hemoglobin levels were significantly elevated in the sequential administration group, suggesting improved tolerance with this regimen (Figure 6F). Additionally, histological HE staining and γ‐H2AX immunohistochemical staining both confirmed the comparable therapeutic efficacy of both strategies (Figure 6G–I), aligning with the in vitro results.

**Figure 7:**
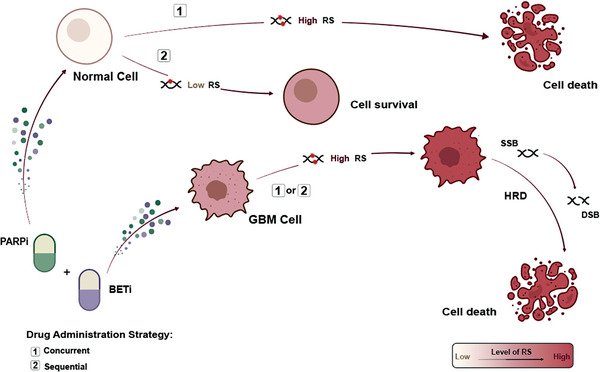
Schematic Summary of the Main Findings in This Study. Compared to concurrent administration, the sequential use of PARPi and BETi selectively targets GBM cells, which have elevated basal replication stress, while sparing normal cells. Additionally, BETi inhibits the HR repair, inducing synthetic lethality in combination with PARPi. The color intensity of the cells indicates the degree of replication stress.

In conclusion, our study leverages the residual effects of PARPi, demonstrating that sequential administration with BETi intensifies replication stress in GBM, leading to subsequent DNA damage. This strategy capitalizes on the inherent differences in baseline replication stress between GBM and normal glial cells, sparing normal glial cells from significant impact. Concurrently, BETi impedes HR repair, thereby hindering the repair of PARPi‐induced DSBs, contributing to the death of GBM cells (**Figure** **7**).

## 3 Discussion

In most oncology clinical trials, effective dosages often come with significant toxicity. A straightforward reduction in dosage, while mitigating adverse effects, usually compromises therapeutic outcomes.^[^ ^23^ ^]^ The balance between efficacy and safety presents a formidable challenge to clinical researchers. It has been nearly two decades since two distinct research groups unveiled the synthetic lethality interaction between PARP inhibitors (PARPi) and BRCA1/2 mutations.^[^ ^24^ ^]^ Despite this progress, the therapeutic indications and spectrum of PARPi have been limited, transitioning from initially targeting BRCAness to now focusing on patients with HRDness characteristics.^[^ ^25^ ^]^ Prolonged administration of PARPi is associated with side effects like nausea, fatigue, and especially anemia, leading to treatment discontinuation.^[^ ^26^ ^]^ Therefore, optimizing therapeutic strategies involving PARPi remains critical in oncology research.

Cancer cells exhibit chronic replication stress due to the loss of proteins that protect or repair stressed replication forks,^[^ ^27^ ^]^ coupled with persistent proliferative signaling, creating a therapeutic vulnerability. This chronic replication stress stands out as a distinctive hallmark of tumor cells.^[^ ^28^ ^]^ With an expanding network of targetable proteins involved in the replication stress response, replication stress has emerged as an enticing clinical target.^[^ ^29^ ^]^ Unlike normal cells, cancer cells can be driven toward death by introducing DNA damage or propelling them into detrimental cell cycle phases associated with unresolved replication stress.^[^ ^30^ ^]^ This understanding paves the way to explore strategies that elevate replication stress in GBM cells to intolerable levels while ensuring it remains sub‐lethal for normal cells.

Our study, driven by data on the transcriptional and proteomic impacts of the BETi Birabresib on GBM, reports a combined sequential inhibition strategy targeting BET and PARP, which reduces toxicity without compromising therapeutic efficacy. The effectiveness of this sequential administration is based on two potential mechanisms: first, by leveraging the residual effects of PARPi, subsequent BETi administration can elevate replication stress levels and lead to the inactivation of GBM cell cycle checkpoints. Prolonged stalling of replication forks can result in their collapse, and DNA replication across SSBs and ssDNA gaps can generate single‐ended DSBs. When two replication forks converge at SSBs or ssDNA gaps, double‐ended DSBs can arise. Second, BETi can also induce HRD in GBM cells. When DSB levels at stalled forks exceed repair capacity, they lead to genomic instability, rendering GBM cells more susceptible to death.^[^ ^27^, ^31^ ^]^ In contrast, sequential treatment in normal glial cells only induces a slight increase in replication stress, without leading to lethal DNA damage levels.

These findings underscore that the appropriate residual effects of PARPi combined with the inherently higher replication stress levels in GBM cells compared to normal glial cells, are crucial for the therapeutic efficacy of sequential PARPi and BETi administration. Further research will also integrate patient‐derived material to significantly enhance the translational impact of our findings. Our study suggests that GBM patients with diverse genetic backgrounds and histology, as well as those previously treated with PARPi, could potentially benefit from this strategy. Future efforts will focus on advancing this therapeutic approach into clinical trials for GBM.

## 4 Experimental Section

### Reagents and Antibodies

Birabresib, JQ1, Olaparib, and Rucaparib were purchased from Selleck Chemicals. The following antibodies were used: Cleaved Caspase‐3 (Cell Signaling Technology, 9664), PARP (Cell Signaling Technology, 9532), γ‐H2AX (Abcam, ab81299), BRD4 (Cell Signaling Technology, 13440), p‐CHK1 (Cell Signaling Technology, 2348), p‐CDC2 (Cell Signaling Technology, 4539), p‐ATR (Cell Signaling Technology, 2853), pH3 (Cell Signaling Technology, 53348), Cyclin B1 (Cell Signaling Technology, 12231), γ‐H2AX (Cell Signaling Technology, 7631), BRCA1 (Cell Signaling Technology, 14823), BRCA2 (Abcam, ab216972), RAD51 (Abcam, 133534), β‐actin (Cell Signaling Technology, 4970), antirabbit IgG, HRP‐linked antibody (Cell Signaling Technology, 7074), and anti‐rabbit IgG (H+L), F(ab')2 Fragment (Alexa Fluor 488 Conjugate, Cell Signaling Technology, 4412).

### Cell Culture

U87 and U251 cells were purchased from the Cell Bank of the Chinese Academy of Sciences (Shanghai, China). Immortalized human glial cells SVGP12 were sourced from the American Type Culture Collection (ATCC). Human primary glial cells HA1800 were purchased from ScienCell. SHG140 and DT001 are primary GBM cells isolated from fresh tumor tissues. Cells were cultured in Dulbecco's Modified Eagle Medium (DMEM) or Roswell Park Memorial Institute 1640 medium (RPMI 1640) supplemented with 10% fetal bovine serum (FBS, Gibco), penicillin (100 U mL^−1^), and streptomycin (100 µg mL^−1^) in a humidified incubator with an atmosphere containing 5% CO_2_ at 37 °C. Cells were passaged routinely every 2–3 days and maintained for a maximum of 20 passages of subcultures.

### Cell Viability Assay and Synergy Assay

Cell viability was measured by CCK‐8 assay as previously described.^[^ ^32^ ^]^ Cells were seeded at a density of 5 × 10^3^ cells mL^−1^ in a volume of 200 µL/well in 96‐well plates. The next day, cells were treated with DMSO or the indicated concentrations of Birabresib and/or Olaparib/Rucaparib. After 48 or 96 h, 10 µL of CCK‐8 (Beyotime) was added to each well. After another 2 h of incubation at 37 °C, the light absorbance was measured at 450 nm using an iMark microplate reader (Bio‐Rad). For the synergy assays, the proliferation of the cells in the different treatment groups was presented as a percentage of control (DMSO‐treated) cells, and the percent of growth inhibition was calculated. ZIP Synergy scores were calculated using the Synergyfinder 3.0.

### Clonogenic Survival Assay

A clonogenic survival assay was performed as previously described.^[^ ^33^ ^]^ Cells were seeded in 24‐well plates at a density of 5 × 10^2^ cells per well with 1 mL medium. After 24 h, cells were treated with the indicated concentrations of Birabresib in combination with Olaparib/Rucaparib for 72 h and were further incubated in a drug‐free medium for 7–10 days to form colonies. The colonies were fixed and stained with 0.25% crystal violet (Sigma‐Aldrich).

### Apoptosis Assay

Cell apoptosis analysis was performed as previously described.^[^ ^33^ ^]^ The cells were treated with 0.5 µm Birabresib and/or 20 µm Olaparib/Rucaparib. After harvesting, the cells were resuspended in 100 µL of binding buffer and incubated with Annexin V/PI solution (Annexin V‐FITC/PI Apoptosis Detection Kit, BD Biosciences) in the dark for 15 min. Samples were acquired on a FACS Verse Flow Cytometer (BD Biosciences) and analyzed using FlowJo Software (BD Life Sciences).

### Alkaline Comet Assay

Alkaline comet assays were performed as previously described.^[^ ^15a^ ^]^ After treatment with 0.5 µm Birabresib and/or 20 µm Rucaparib, cells (1 × 10^5^ mL^−1^) were collected and mixed with low melting point agarose at a ratio of 1:10 (v/v), and 50 µL of the cell suspension was immediately added onto comet slides. The slides were then incubated at 4 °C for 10 min, immersed in lysis solution for 30 min, and in an alkaline unwinding solution for 20 min in the dark. Following electrophoresis, the cells were stained with SYBR Gold (Invitrogen). The quantification of tail DNA was performed with CASP software (CaspLab).

### Immunofluorescence Staining

Immunofluorescence staining was performed as previously described.^[^ ^15a^ ^]^ Cells were seeded onto coverslips in 24‐well plates at a density of 5 × 10^3^ cells per well in 1 mL of medium. After 24 h, cells were treated with Birabresib, Rucaparib, or a combination for indicated durations. Cells were fixed in 4% paraformaldehyde and permeabilized with 0.2% Triton X‐100 in PBS. The cells were then blocked in 5% donkey serum in the presence of 0.1% Triton X‐100 and stained with the γ‐H2AX, pH3, or RAD51 primary antibody, then incubated with the secondary antibody coupled to Alexa Fluor 488. After being rinsed and washed three times with PBS, the slides were mounted with VECTASHIELD mounting medium (Vector Laboratories) containing DAPI. Cells were observed using a BX51 fluorescence microscope (Olympus).

### siRNA Knockdown

siRNA knockdown of BRD4 was performed as previously described.^[^ ^34^ ^]^ siRNA for BRD4 (5′‐ GCCAAATGTCTACACAGTATA‐3′) was purchased from Sigma‐Aldrich. Briefly, cells were plated in six‐well plates and transfected with 30 nm of BRD4‐targeting or nontargeting siRNA (siNT) using Lipofectamine 3000 transfection reagent (Invitrogen) according to the manufacturer's protocol.

### qRT‐PCR

qRT‐PCR was performed as previously described.^[^ ^35^ ^]^ RNA was isolated using an RNeasy Mini kit (Qiagen) and used to synthesize complementary DNA (cDNA) using a cDNA Synthesis Kit (GenStar). RT‐PCR was performed with aliquots of cDNA samples mixed with SYBR Green Master Mix (Applied Biosystems). Reactions were performed in triplicates. The fold differences in transcripts were calculated using the ΔΔCt method, and 18S rRNA was used as a control to normalize RNA expression. The primers used are shown in Table S1 (Supporting Information).

### Western Blots

Western blots were performed as previously described.^[^ ^36^ ^]^ Cells were seeded in 6‐well plates at a density of 2 × 10^5^ cells per well in 2 mL medium. After 24 h, the cells in each well were treated with either DMSO, Rucaparib, or Birabresib, or subjected to concurrent and sequential treatments. After that, cells were lysed, and total proteins were harvested. Equal amounts of protein (20‐50 µg) were separated using 8% or 12% SDS‐PAGE and transferred to polyvinylidene difluoride membranes (Bio‐Rad). After blocking in 5% nonfat dry milk, the membranes were incubated with appropriate primary antibodies overnight at 4 °C, washed, and incubated with respective HRP‐conjugated secondary antibodies for 1 h at room temperature. The signals were detected using a ChemiDoc XRS+ System (Bio‐Rad) after exposure to chemiluminescence reagents (Bio‐Rad), and β‐actin was used as the loading control.

### Cell Cycle Analysis

Cell cycle analysis was performed as previously described.^[^ ^37^ ^]^ Cells were seeded into six‐well plates and treated with indicated drugs. After 12 or 24 h of incubation, cells were collected, suspended in PBS, and fixed in 75% ethanol at 4 °C overnight. Next, cells were incubated with anti‐phospho‐Histone H3 (Ser10) for 1 h. Then washed and resuspended in PBS containing 50 µg mL PI and 100 µg mL RNase (Solarbio). All samples were analyzed on a FACS Verse Flow Cytometer (BD Biosciences).

### DR‐GFP Reporter Assay

HR efficiency was evaluated as previously described.^[^ ^38^ ^]^ The HR repair reporter substrate pDR‐GFP plasmid and the I‐SceI‐expression plasmids/empty vectors were co‐transfected into cells using Lipofectamine 3000 (Invitrogen). GFP‐expressing plasmid (pEGFP‐C1) was used as a transfection efficiency control. Birabresib or Rucaparib was added 6 h after the transfection. After 48 h, GFP‐positive cells were detected on a FACS Verse flow cytometer (BD Biosciences).

### DNA Fiber Assay

DNA fiber assay was performed as previously described.^[^ ^39^ ^]^ The cells were treated with Birabresib and Rucaparib, or their concurrent or sequential combinations. After removing the drugs, cells were starved for 2 h and then labeled with 25 mm CldU (1:1000 dilution of 25 mm stock solution) for 30 min at 37 °C. Cells were subsequently washed once with PBS and labeled with 250 mm IdU (1:200 dilution of 50 mm stock solution) for another 30 min at 37 °C. Cells were collected with trypsin and resuspended at a concentration of 1 × 10^6^ cells mL in ice‐cold PBS. For each sample, 200 µL of lysis buffer (200 mm Tri‐HCl pH 7.4, 50 mm EDTA, 0.5% SDS) was added and mixed, followed by a 5 min lysis on ice. Next, 10 µL of the suspension was transferred to a microscope slide, allowing the DNA to flow gently down the slide. After air‐drying, slides were fixed in methanol/acetic acid (3:1) for 10 min. Slides were then washed with H_2_O, air‐dried, and denatured in 2N HCl for 30 min. After washing with PBS, slides were incubated in blocking solution (PBS containing 5% BSA) for 30 min, followed by a 2‐h incubation at room temperature with rat anti‐BrdU antibody [clone BU1/75 (ICR1), Abcam] at a 1:60 dilution and mouse anti‐BrdU antibody [clone B44, BD Biosciences] at a 1:100 dilution. After washing with PBS, slides were fixed in 4% paraformaldehyde for 10 min. Following another PBS wash, slides were incubated with antirat AlexaFluor 488 antibody and anti‐mouse AlexaFluor 568 antibody, both at a 1:300 dilution, for 1 h at room temperature. After the final wash, slides were mounted in Fluoromount‐G and observed under a 40× objective on an Olympus BX53 microscope. The lengths of the red and green labeled patches were measured using ImageJ software (National Institutes of Health; http://rsbweb.nih.gov/ij/) and converted to micrometers.

### Orthotopic Zebrafish Xenograft Model

Xenotransplantation of human GBM U87 cells and proliferation assessment were performed as previously described.^[^ ^19^ ^]^ Wild‐type AB zebrafish (Danio rerio) were maintained and reared under a 14 h light and 10 h dark cycle at 28 °C in a controlled multi‐tank recirculating system. Embryos were collected and incubated in reconstituted water (60 µg mL^−1^ sea salt in RO water with 1 ppm methylene blue). At 48 h postfertilization, embryos were anesthetized using 1.2 mm tricaine and moved onto a modified agarose gel mold for tumor cell microinjection. A total of 50–100 U87‐RFP cells suspended in 5 nL of serum‐free culture medium were injected into the brains of zebrafish larvae using a pneumatic pico‐pump injector. After cell implantation, injected embryos were screened and separately transferred to a 48‐well plate containing drugs in 2 mL of E3 media and incubated at 32 °C for 4 days. Xenografts were observed under an inverted microscope (IX71, Olympus) every 2 days. The fluorescence intensity of xenografts was quantified with ImageJ software.

### Orthotopic Nude Mouse Xenograft Model

The murine intracranial tumor model was generated as previously described.^[^ ^19^ ^]^ U87‐Luc cells (2.0 × 10^5^ cells per mouse) were injected into the right striatum of 4‐week‐old male BALB/c nude mice. Ten days after injection (defined as day 0), tumors were measured using an IVIS luminescent imaging system (IVIS Spectrum, PerkinElmer) and randomly assigned to the following treatment groups: vehicle, Rucaparib (10 mg kg^−1^, i.p.), Birabresib (50 mg kg^−1^, i.p.), concurrent (Rucaparib + Birabresib), and sequential (Rucaparib 7 days + Birabresib 7 days) groups. On days 7,14, and 21 animals were examined by luminescence imaging. The mice were euthanized on day 21 to collect tumor samples. Data were analyzed using in vivo imaging software (Living Image 4.5.2, PerkinElmer) and normalized to the initial postinjection signal on day 0.

### H&E and Immunohistochemical (IHC) Staining

H&E and IHC staining were performed as previously described.^[^ ^40^ ^]^ H&E staining was used to detect pathological changes in morphology. Apoptotic cells in tumor tissues were stained using a TUNEL Apoptosis Detection Kit (Beyotime). For immunohistochemical analysis, formalin‐fixed, paraffin‐embedded tumors were sectioned, and slides were deparaffinized using xylene (Thermo Fisher). Endogenous peroxidases were quenched with 3% hydrogen peroxide in methanol. Staining was performed using antibodies against γ‐H2AX (1:50). Counterstaining was performed with Mayer's hematoxylin (Dako). Slides were observed under an Olympus CX21 microscope and scanned with a high‐resolution digital slide scanner (Pannoramic 250, 3DHistech) to capture images.

### RNA‐seq Profiling and Analysis

RNA‐seq was generated as previously described.^[^ ^19a^ ^]^ U87 cells were treated with Birabresib (0.5 µm) or DMSO for 24 h, and total RNA was isolated using TRIzol reagent. RNA concentrations were quantified using a NanoDrop Spectrophotometer (Thermo Fisher), and RNA integrity was assessed using the RNA Nano 6000 Assay Kit on a Bioanalyzer 2100 system (Agilent Technologies). Only samples with RIN values of >6.0 were used for experiments. A complementary DNA library was prepared, and sequencing was performed according to the Illumina standard protocol by Beijing Novel Bioinformatics Co., Ltd. (https://en.novogene.com/). Specifically, cDNA libraries were prepared using an Illumina NEBNext UltraTM RNA Library Prep Kit. After cluster generation, the library preparations were sequenced on an Illumina NovaSeq 6000 platform, and 150 bp paired‐end reads were generated. For the data analysis, raw data (raw reads) in fastq format were first processed through in‐house Perl scripts. Clean reads were obtained by removing reads containing adapters, poly Ns, and low‐quality reads from raw data. Reference genome and gene model annotation files were downloaded from the genome website directly. The index of the reference genome was built using Hisat2 v2.0.5, and paired‐end clean reads were aligned to the reference genome using Hisat2 v2.0.5. Mapped reads of each sample were assembled using StringTie (v1.3.3b) in a reference‐based approach. Feature Counts v1.5.0‐p3 were used to quantify the read numbers mapped to each gene. Differential expression analysis was conducted using DESeq2 (v. 1.38.3). Differentially Expressed Genes (DEGs) with a *p*‐value < 0.05 were visualized as a volcano plot using the ggplot2 package (v. 3.4.2) and the ggrepel package (v. 0.9.3). Heatmaps were generated using the pheatmap package (v.1.0.12). After converting the official gene symbol IDs to Entrez IDs using the org.Hs.eg.db package (v.3.16.0), DEGs were mapped to KEGG (Kyoto Encyclopedia of Genes and Genomes) and GO (Gene Ontology) pathways using the clusterProfiler package (v.4.6.2). For Gene Set Enrichment Analysis (GSEA), each gene's fold change (FC) between subtypes was first calculated, followed by descending sorting of the input genes based on FC values. GSEA was performed using the xPierGSEA function in the Pi package (v.2.10.0), and related plots, including specific genes showing the leading edge, were drawn using the xGSEA dot plot function. Gene Set Variation Analysis (GSVA) was conducted using the GSVA package (v.1.46.0), and results were presented as a volcano plot. All analyses were performed in R v.4.2.4.

### Protein Extraction and Digestion

U87 cells were seeded in six‐well plates at a density of 2 × 10^5^ cells per well in 2 mL of medium, and incubated for 24 h, followed by treatment with DMSO (Control) or Birabresib (0.5 µm). The treated cells were lysed, and total proteins were harvested. 50 µg of protein sample was denatured by adding one‐fourth 8 m urea (Aladdin). Then, 50 mm of ammonium bicarbonate (Aladdin) was added to make the total volume to 100 µL. Subsequently, the protein sample was exposed to 1 µL of 200 mm Dithiothreitol (DTT) (Thermo) for 30 min at 60 °C, and then alkylated with 1 µL of 500 mm iodoacetamide (Aladdin). After digestion with trypsin (Thermo) (4 µL, 0.25 µg µL) at 37 °C overnight, the samples were desalted with a C18 SPE column (Millipore) and vacuum‐dried, to be available for MS analysis.

### LC‐MS/MS Analysis

LC‐MS/MS Analysis was performed as previously described.^[^ ^19b^ ^]^ The digested peptide samples were analyzed using a Q Exactive Plus mass spectrometer and an EASY nano‐liquid chromatography (EASY nLC 1200, Thermo Scientific) with an EASY nanoelectrospray interface. The nano liquid chromatography system was equipped with a Thermo Scientific Acclaim Pepmap nano‐trap column (C18, 5 µm, 100 Å, 100 µm × 2 cm) and a Thermo Scientific EASY‐Spray column (Pepmap RSLC, C18, 2 µm, 100 Å, 50 µm × 15 cm). Solvent A (0.1% formic acid) and solvent B (80% CH3CN/0.1% formic acid), whose gradients were as follows: 0–8% B for 3 min, 8–28% B for 42 min, 28–38% B for 5 min, 38–100% B for 10 min, were used for the nano liquid chromatography analysis. The mass spectra were searched against the UniProt database, and the MS raw data for each sample were searched using Maxquant (Version 2.0.3.1). Related parameters and instructions were as follows: samples with carbamidomethylation of cysteine were set as a fixed modification. Oxidation (M) was set as the variable modification. Searches were performed with trypsin cleavage specificity allowing two miscleavage events. The precursor mass tolerance was set to 10 parts‐per‐million (ppm) and a fragment mass tolerance of 0.02 Da. A maximum false discovery rate (FDR) of 1.0% was set for protein and peptide identification. Protein identification was based on at least one unique peptide identification. Protein quantification was calculated as the median of unique peptides of the protein.

### Proteome and Transcriptome‐Proteome Correlation Analysis

The methods for differential analysis and plotting for the proteome were the same as for the transcriptome, except that the analysis in R utilized the limma package (v.3.44.3). Protein interaction analysis was performed using the Cytoscape software (v.3.10.0), where differentially expressed proteins were mapped to Reactome (a biological pathways database) using the stringApp plugin (v.2.0.1), and differential proteins included in key pathways were selected to form subnetworks. The ggplot2 package (v. 3.4.2) was used to visualize co‐expressed DEGs of the proteome and transcriptome as a nine‐quadrant plot. The VennDiagram package (v. 1.7.3) was employed to plot a Venn diagram of all DEGs from the proteome and transcriptome. All analyses involving software packages were conducted in R v.4.2.4.

### Statistical Analysis

Data from three independent experiments are presented as mean ± SEM. Unpaired, two‐tailed Student's *t*‐test was used for two‐group comparisons. ANOVA with Bonferroni's correction was used to compare multiple groups. A *p*‐value <0.05 was considered statistically significant. Before statistical analysis, variations within each group and the assumptions of the tests were assessed.

## Conflict of Interest

The authors declare no conflicts of interest.

## Author Contributions

X.P., X.H., and S.Z. contributed equally to this work. X.P., X.H., S.Z., N.Z,. S.H., Y.W., and Z.Z. performed the experiments. S.Z., H.G., Z.Y., X.Y., Z.T., Y.D., Z.Z., X.C., F.X.C., and M.E. analyzed the data. X.P., N.J., Y.Z., and D.K. designed the experiments. X.P. and D.K. wrote the main manuscript. X.P., N.J., Y.Z., and D.K. revised the manuscript. All authors reviewed the manuscript.

## Supporting information

Supporting Information

Supplemental Table 1

Supplemental Table 2

## Data Availability

The data that support the findings of this study are available in the supplementary material of this article.
