# Supplementary material for: Sequential Inhibition of PARP and BET as a Rational Therapeutic Strategy for Glioblastoma
Source: Adv Sci (Weinh). 2024 Jun 19;11(30):2307747. doi: 10.1002/advs.202307747 (PMC11321613; doi:10.1002/advs.202307747)
Supplement: Supplementary file 1 — Supporting Information [file ADVS-11-2307747-s001.docx]

**List of Items in Supplementary Material**

| **Figure S1** | **Enrichment Analysis Results from Other Databases and RT-PCR Validation of Corresponding Key Genes, Related to Figure 1.** |
| --- | --- |
| **Figure S2** | **Quantitative Proteomics Reveals Differentially Expressed Proteins, Related to Figure 1.** |
| **Figure S3** | **ZIP Model Synergy Test in GBM Cells, Related to Figure 2.** |
| **Figure S4** | **Inhibition of BRD4 at Both the Genetic and Pharmacological Levels Enhances the Cytotoxic Effect of PARPi on GBM, Related to Figure 2.** |
| **Figure S5** | **Cell Cycle and DNA Damage-Related Experiments in Established GBM Cell Lines (U251 and U87) and Patient-Derived Primary GBM Cell Line (SHG140), Related to Figure 3.** |
| **Figure S6** | **Sequential Treatment with Rucaparib and Birabresib in Patient-Derived Primary GBM Cell Line (SHG140), Related to Figure 4.** |
| **Figure S7** | **Toxicity Assessment of Sequential Treatment with Rucaparib and Birabresib, Related to Figure 6.** |
| **Table S1** | **The Primer Sequences Used in This Study.** |
| **Table S2** | **The List of DEGs from Transcriptomic Analysis and DEPs from Proteomic Analysis.** |
| **Table S3** | **The List of GSEA and GSVA Results.** |

**
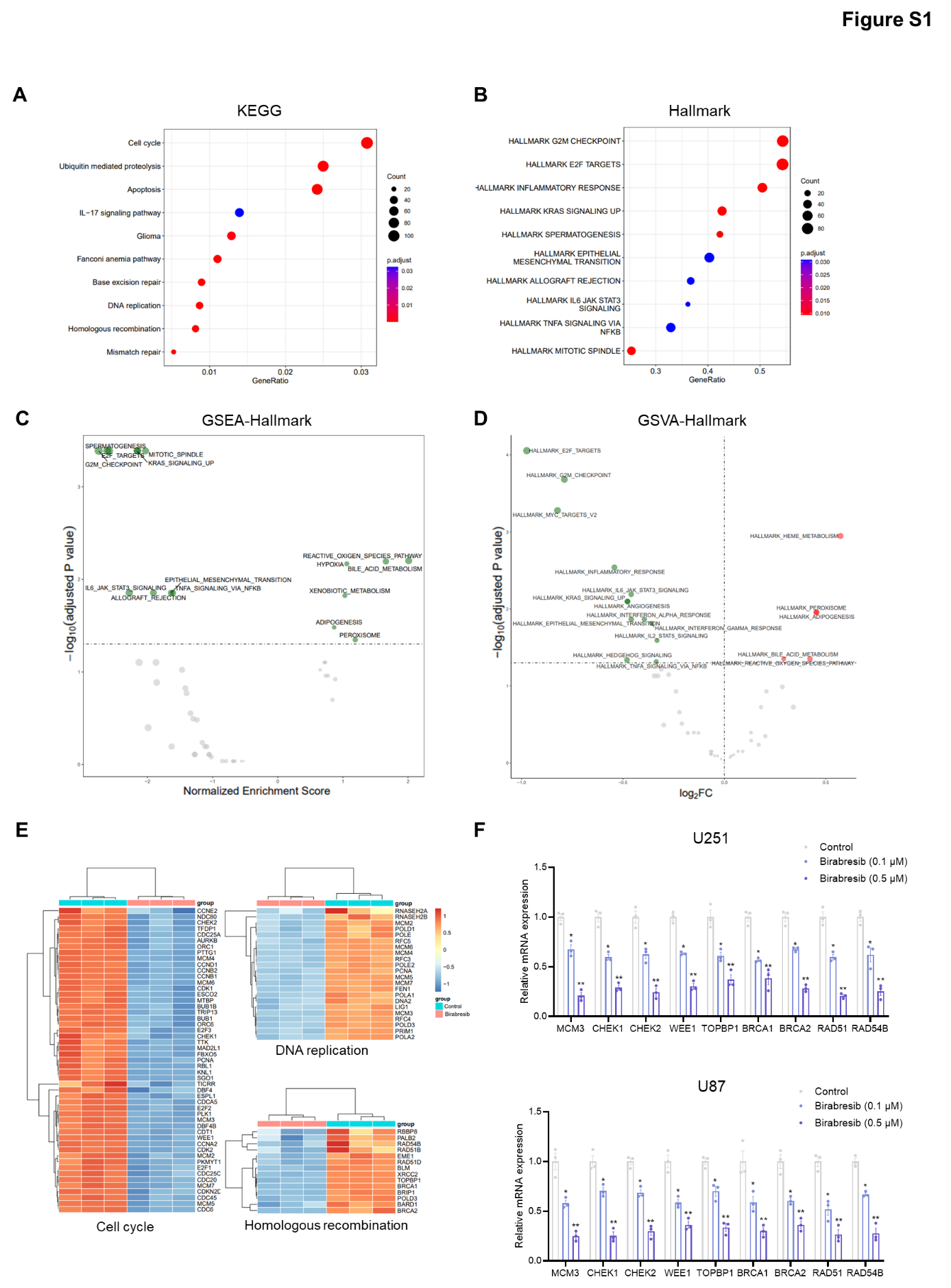
**

**Figure S1. Enrichment Analysis Results from Other Databases and RT-PCR Validation of Corresponding Key Genes, Related to Figure 1.**

(**A, B**) Bubble charts illustrating the enrichment of differentially expressed genes mapped to KEGG and Hallmarks pathways, including pathway term names and gene ratios. Adjusted P-values are represented by color variations, and the size of the bubbles corresponds to the number of genes in each term. (**C**) Scatter plot showing gene set enrichment analysis of differentially expressed genes mapped to Hallmarks pathways, including normalized enrichment scores and adjusted P-values. The horizontal dashed line indicates an adjusted P-value of 0.05. (**D**) Volcano plot illustrating gene set variation analysis of genes mapped to Hallmarks pathways, including log-fold changes in enrichment scores and adjusted P-values. The horizontal dashed line indicates an adjusted P-value of 0.05. (**E**) Heatmap displaying genes from specific Reactome pathways, with FPKM values converted to Z-scores and subjected to clustering analysis. (**F**) qRT-PCR analysis of U251 and U87 cells treated with Birabresib for 24 h to quantify representative DNA repair-related genes detected in RNA-seq data. 18S rRNA was used for normalization. Graphs are presented as the mean ± SEM from three independent experiments; P-values were determined using a two-tailed unpaired Student’s t-test; *p < 0.05; **p < 0.01; ***p < 0.001; ****p < 0.0001.


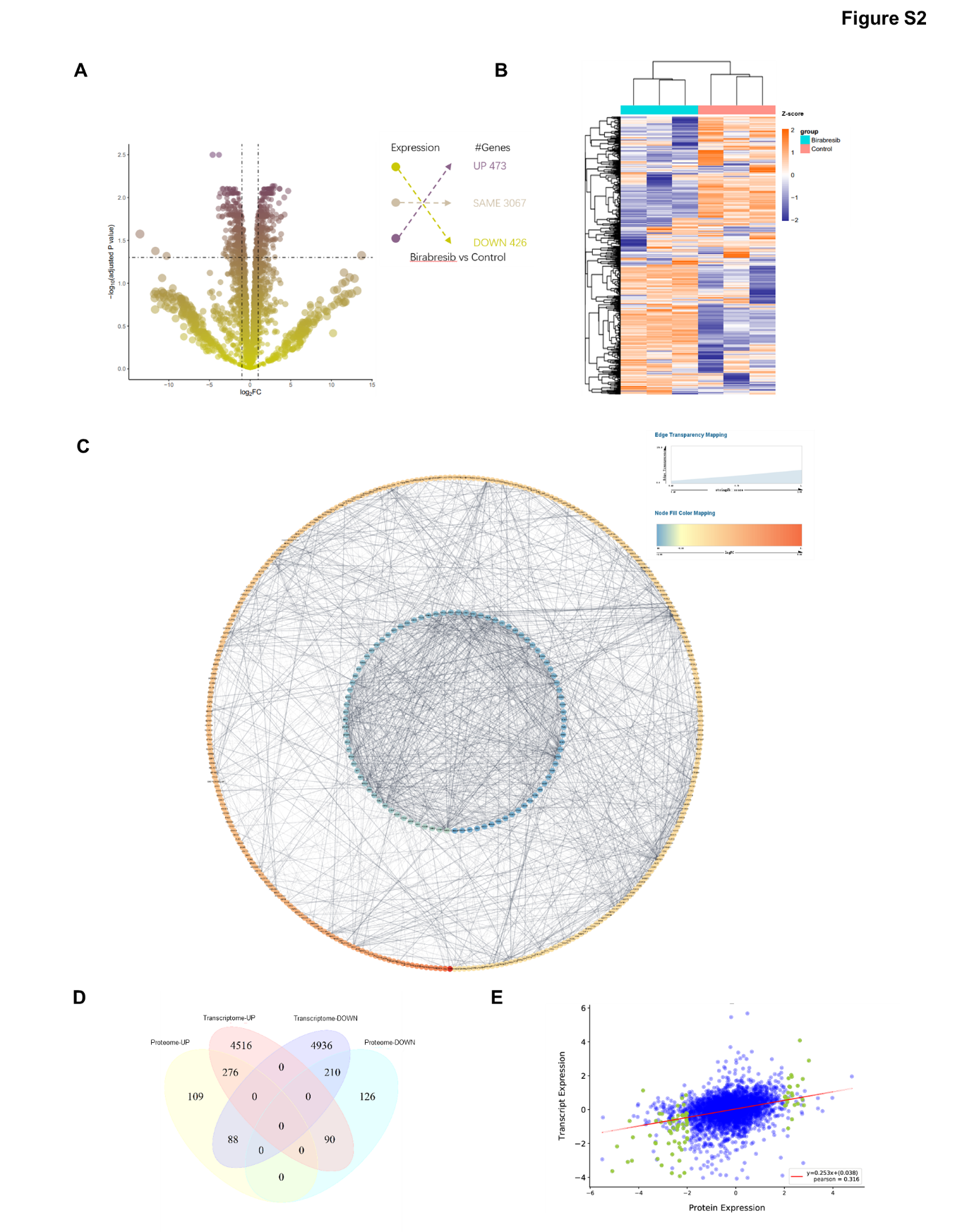


**Figure S2. Quantitative Proteomics Reveals Differentially Expressed Proteins, Related to Figure 1.**

(**A**) Volcano plot displaying global protein expression, showing log-fold changes in protein expression and adjusted P-values. The horizontal dashed line indicates an adjusted P-value of 0.05. (**B**) Heatmap showing differentially expressed proteins, with protein expression levels converted to Z-scores and subjected to clustering analysis. (**C**) Protein-protein interaction network diagram encompassing overall differentially expressed proteins. The outer circle represents upregulated proteins, and the inner circle represents downregulated proteins. Log-fold changes in protein expression are depicted by color variations in a clockwise decreasing manner, and protein interaction composite scores are represented by edge transparency. (**D**) Venn diagram presenting the overlap of differentially expressed genes in the transcriptome and proteome. (**E**) Scatter plot presenting the correlation between protein expression and gene expression, the correlation is represented by the Pearson correlation coefficient. Graphs are presented as the mean ± SEM from three independent experiments; P-values were determined using a two-tailed unpaired Student’s t-test; *p < 0.05; **p < 0.01; ***p < 0.001; ****p < 0.0001.


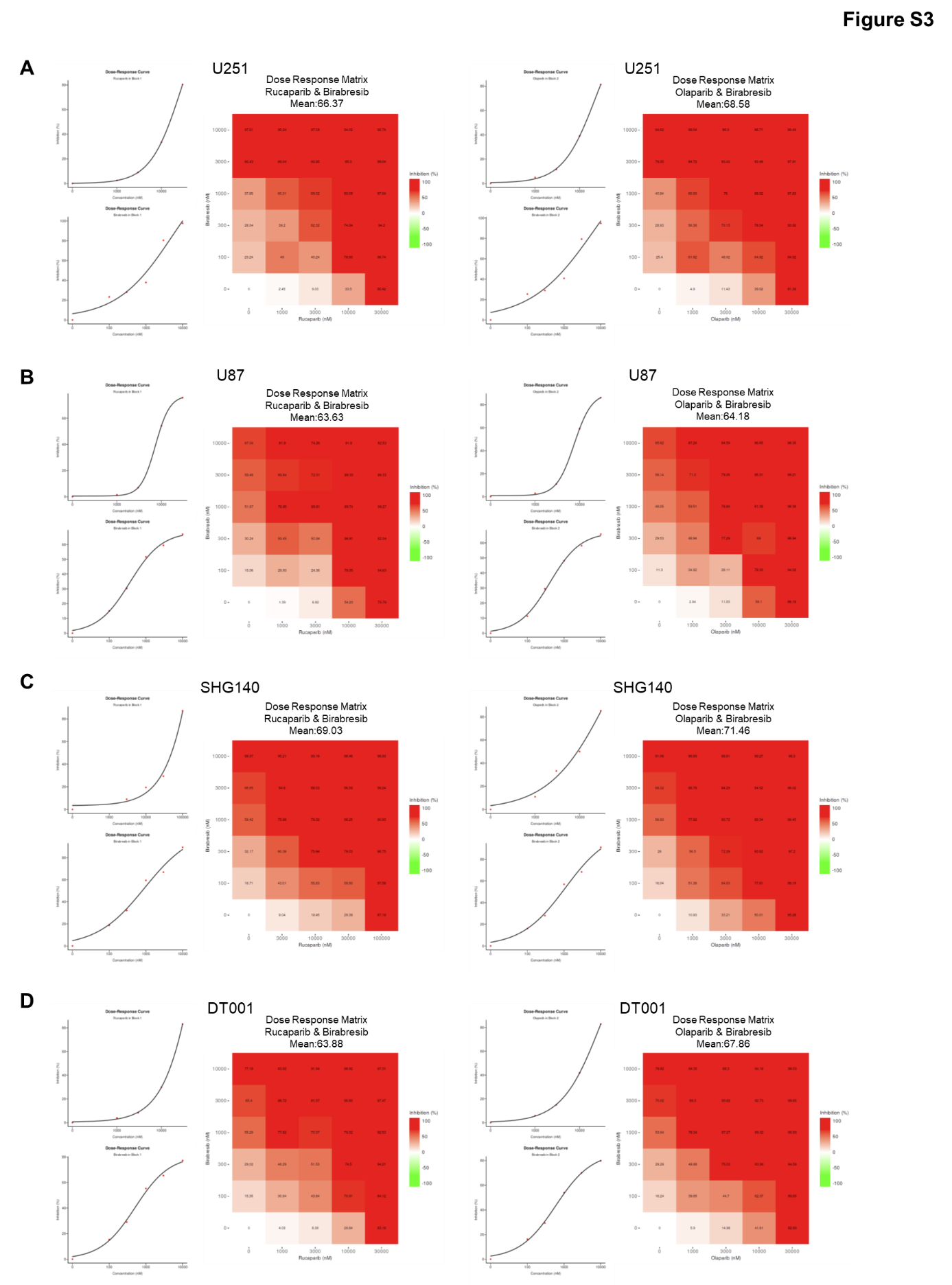


**Figure S3. ZIP Model Synergy Test in GBM Cells, Related to Figure 2.**

Dose-response curve and Dose-response matrix of Olaparib/Rucaparib and Birabresib in established GBM cell lines U251 and U87 (**A, B**), and patient-derived primary GBM cell lines SHG140 and DT001 (**C, D**). Graphs are presented as the mean ± SEM from three independent experiments; P-values were determined using a two-tailed unpaired Student’s t-test; *p < 0.05; **p < 0.01; ***p < 0.001; ****p < 0.0001.


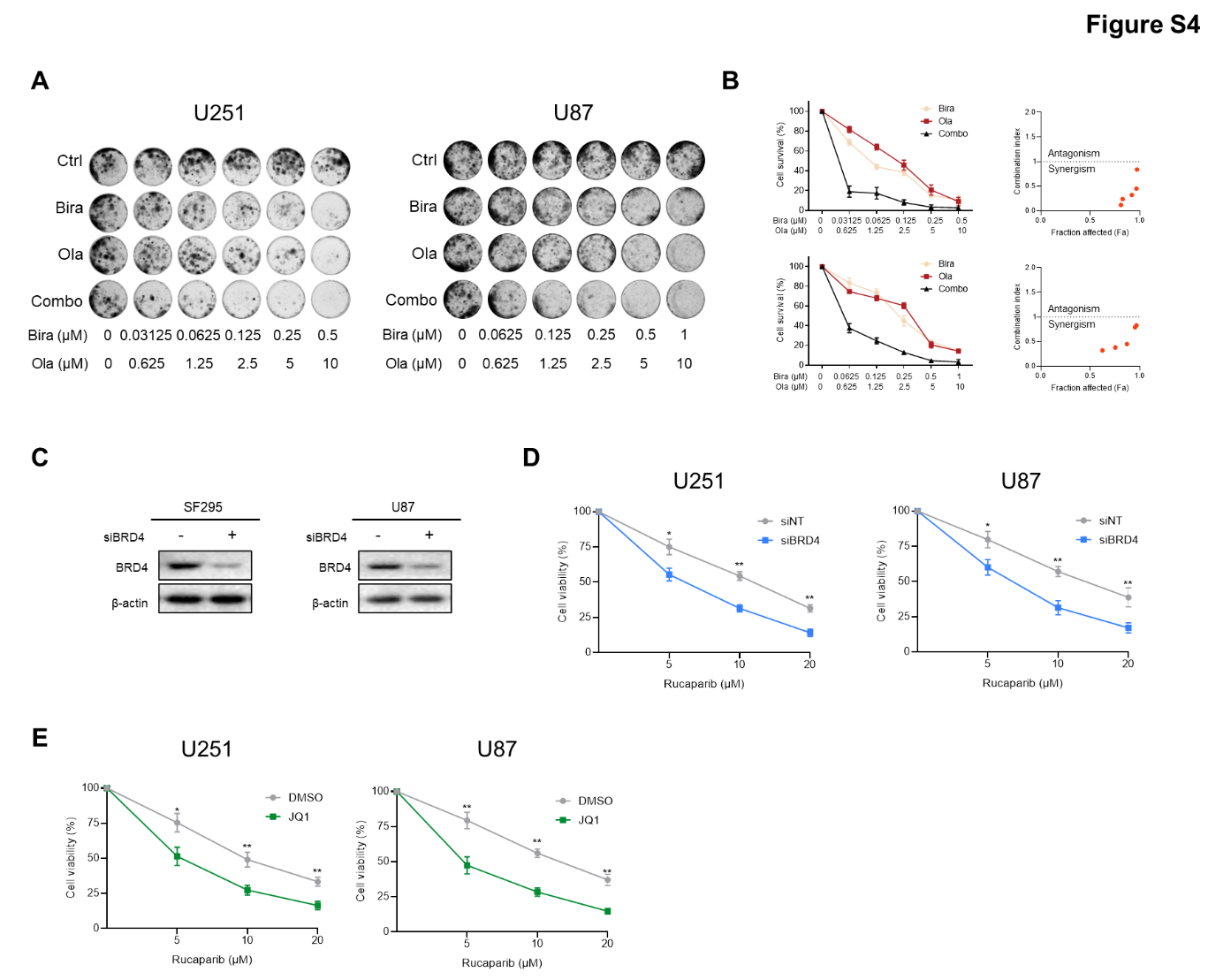


**Figure S4. Inhibition of BRD4 at Both the Genetic and Pharmacological Levels Enhances the Cytotoxic Effect of PARPi on GBM, Related to Figure 2.**

(**A**) Clonogenic assays for GBM cell lines treated with Birabresib and/or Olaparib for 72 h. The cells were recovered for 10-15 days and then subjected to crystal violet staining. (**B**) Quantification of (**A**), the absorbance at 570 nm was measured after incubation with 1% SDS for 3 h. Cell survival (%) is expressed as a percentage of the control; CIs were calculated with CalcuSyn. CI ≤ 0.9 represents synergism, 0.9 < CI ≤ 1.1 represents additivity, and CI > 1.1 represents antagonism. (**C**) Western Blot confirms the knockdown of BRD4 in GBM cells using siRNA. (**D**) Cell Viability Assay assessed the response of GBM cells to Rucaparib with and without BRD4 knockdown. (**E**) The effect of JQ1 on the sensitivity of GBM cells to Rucaparib was investigated using a Cell Viability Assay. Graphs are presented as the mean ± SEM from three independent experiments; P-values were determined using a two-tailed unpaired Student’s t-test; *p < 0.05; **p < 0.01; ***p < 0.001; ****p < 0.0001.


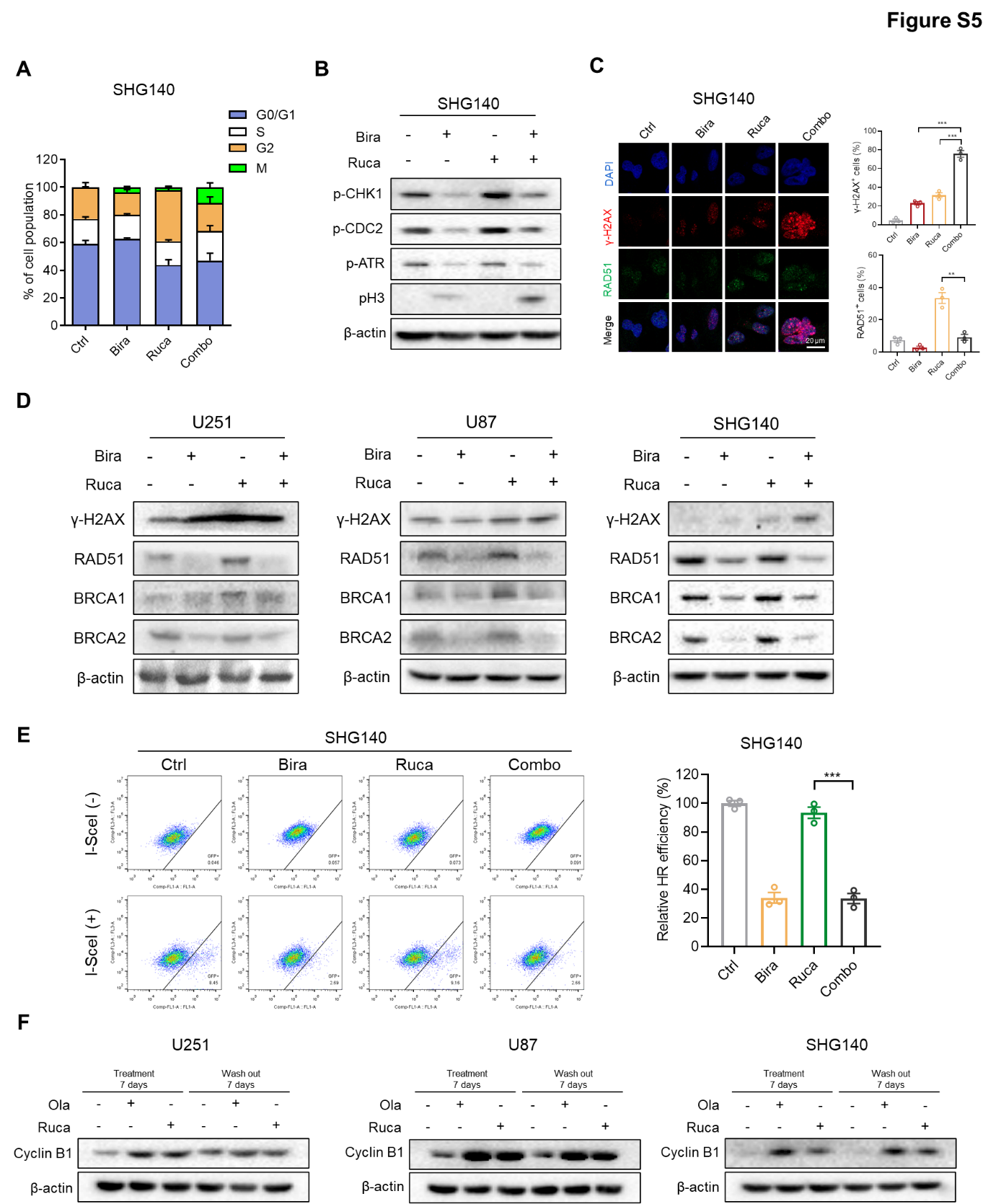


**Figure S5. Cell Cycle and DNA Damage-Related Experiments in Established GBM Cell Lines (U251 and U87) and Patient-Derived Primary GBM Cell Line (SHG140), Related to Figure 3.**

(**A**) SHG140 cells were treated with DMSO, Birabresib, Rucaparib, or a combination of both for 24h, followed by pH3 and PI flow cytometry analysis. (**B**) Expression of the indicated cell cycle checkpoint proteins in SHG140 cells was examined by Western blot. (**C**) Foci assays for γ-H2AX and RAD51 were conducted on U251 and U87 cells that were either untreated (control) or treated with Rucaparib, Birabresib, or their combination. Scale bar, 20 μm. Representative images and quantification of the number of γ-H2AX/RAD51 positive U251 and U87 cells (with > 5 foci per cell) are shown. (**D**) Western blot analysis revealed alterations in proteins associated with HR repair. (**E**) DR-GFP reporter assays evaluated the effect of Birabresib and Rucaparib on HR repair efficiency in SHG140 cells. Relative HR efficiency is expressed as % of the control. (**F**) GBM cells were treated with Olaparib, Rucaparib or DMSO for 7 days, then washed out and re-cultured for 7 days, and western blotted with Cyclin B1. Graphs are presented as the mean ± SEM from three independent experiments; P-values were determined using a two-tailed unpaired Student’s t-test; *p < 0.05; **p < 0.01; ***p < 0.001; ****p < 0.0001.


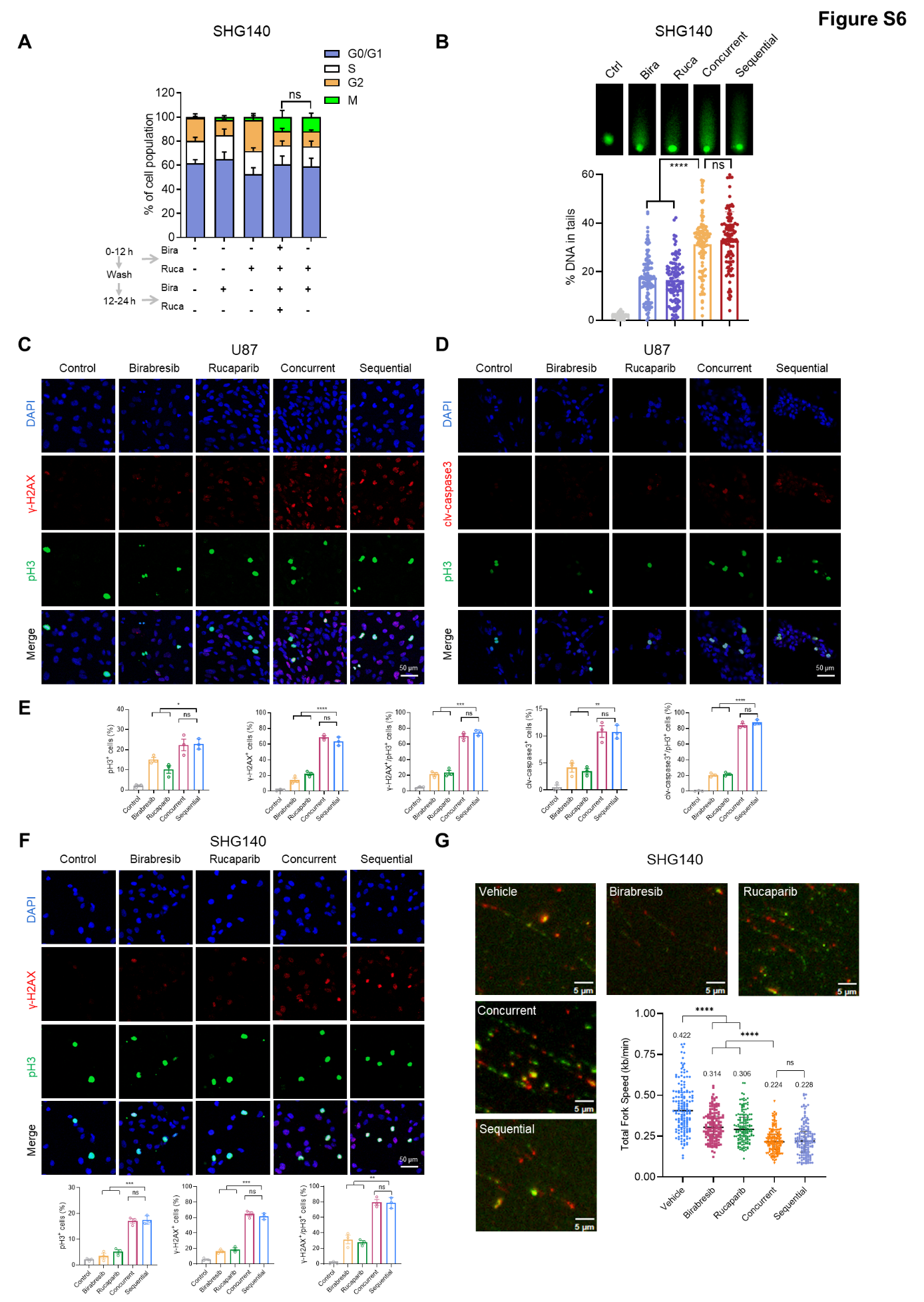


**Figure S6. Sequential Treatment with Rucaparib and Birabresib in Patient-Derived Primary GBM Cell Line (SHG140), Related to Figure 4.**

(**A**) SHG140 cells were treated with DMSO, Birabresib, Rucaparib, sequential, and concurrent treatment, followed by pH3 and PI flow cytometry analysis. (**B**) SHG140 cells were treated as in (**A**) and subjected to Comet assay. DNA damage is quantified as percentage DNA in tail. (**C**) Representative images of U87 cells treated as in (**A**) stained for γ-H2AX, pH3, and DAPI. Scale bar, 50 μm. (**D**) Representative images of U87 cells treated as in (**A**) stained for cleaved caspase-3, pH3, and DAPI. Scale bar, 50 μm. (**E**) Quantitative analysis of (**C**) and (**D**) was performed on five photographs from each of the three independent experiments. (**F**) Representative images of SHG140 cells treated as in (**A**) stained for γ-H2AX, pH3, and DAPI. Scale bar, 50 μm. Quantitative analysis was performed on five photographs from each of the three independent experiments. (**G**) Representative images of GBM cells treated as in (**A**) and subjected to DNA fiber analysis. Scale bar, 10 μm. Mean fork speed (kb/min) is indicated. Graphs are presented as the mean ± SEM from three independent experiments; P-values were determined using a two-tailed unpaired Student’s t-test; *p < 0.05; **p < 0.01; ***p < 0.001; ****p < 0.0001.

**
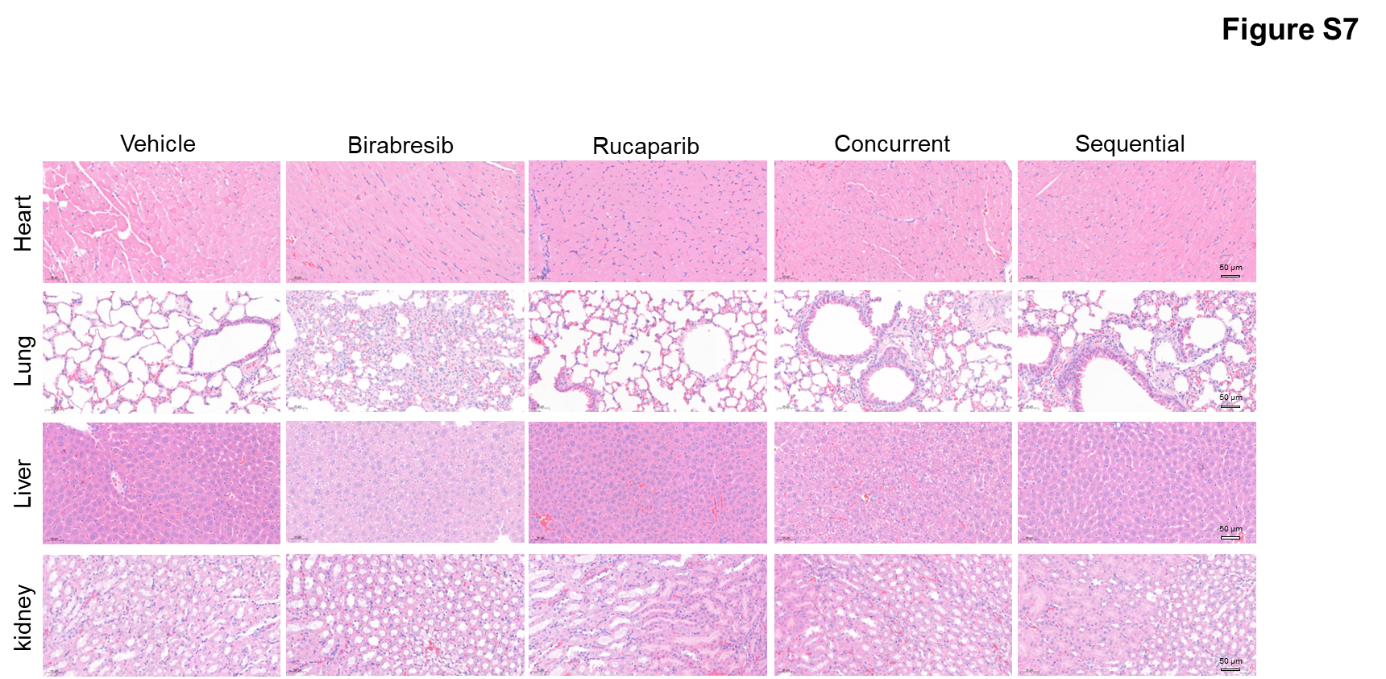
**

**Figure S7. Toxicity Assessment of Sequential Treatment with Rucaparib and Birabresib, Related to Figure 6.**

The major organs (heart, lung, liver, and kidney) of mice in the vehicle, Rucaparib (10 mg/kg), Birabresib (50 mg/kg), concurrent (Rucaparib + Birabresib), and sequential (Rucaparib 7 days + Birabresib 7 days) groups were harvested on the 21st day and analyzed using H&E staining. Representative microphotographs of histological sections are presented.

**Table S1. The Primer Sequences Used in This Study.**

| Genes | Sequence（5’-3’） |
| --- | --- |
| MCM3 | Forward: CCAAGAAGAAAAAGGAGAAGATGGT  Reverse: GTGTTCGGGCTGTAACTGGA |
| CHEK1 | Forward: GACTGGGACTTGGTGCAAAC  Reverse: TGCCATGAGTTGATGGAAGA |
| CHEK2 | Forward: TTATCTGCCTTAGTGGGTATCCA  Reverse: CTGTCGTAAAACGTGCCTTTG |
| WEE1 | Forward: GCGTGGTAGCACACATCATT  Reverse: GTGCAATCACGGCTCTGTAG |
| TOPBP1 | Forward: GAGACTTTGTCCCACAGGGTCCA  Reverse: AACCTTGTGCTCAGGCTCCTGTTA |
| RAD54B | Forward: TCCAGGTCTGAATGAAGAGATTAC  Reverse: TCTAGTACTTTCTTCACTAGGCAG |
| BRCA1 | Forward: GAACGGGCTTGGAAGAAAAT  Reverse: GTTTCACTCTCACACCCAGA |
| BRCA2 | Forward: CAGGTAGACAGCAGCAAGCA  Reverse: AAGCCCCTAAACCCCACTTC |
| RAD51 | Forward: CAGATGCAGCTTGAAGCAAA  Reverse: TTCTTCACATCGTTGGCATT |
| 18S rRNA | Forward: CAGCCACCCGAGATTGAGCA  Reverse: TAGTAGCGACGGGCGGTGTG |

**Table S2. The List of DEGs from Transcriptomic Analysis and DEPs from Proteomic Analysis.**

**Table S3. The List of GSEA and GSVA Results.**
